# Exploring the Utility of *Prunus mahaleb* Extracts as a Source of Natural Bioactive Compounds for Functional Applications

**DOI:** 10.1002/fsn3.70121

**Published:** 2025-03-28

**Authors:** Bayram Atasagun, Ahmet Uysal, Noha Fathallah, Omayma Eldahshan, Abdel Nasser Singab, Mehmet Veyis Cetiz, Gokhan Zengin

**Affiliations:** ^1^ Department of Medical Services and Techniques Vocational School of Health Services, Selcuk University Konya Türkiye; ^2^ Department of Pharmacognosy and Medicinal Plants, Faculty of Pharmacy Future University in Egypt Cairo Egypt; ^3^ Department of Pharmacognosy, Faculty of Pharmacy Ain Shams University Cairo Egypt; ^4^ Faculty of Pharmacy, Center for Drug Discovery Research and Development Ain Shams University Cairo Egypt; ^5^ Department of Medical Biochemistry, Faculty of Medicine Harran University Sanliurfa Turkey; ^6^ Department of Biology, Science Faculty Selcuk University Konya Turkey

**Keywords:** antimutagenicity, antioxidant, enzyme inhibition, health‐promoter, natural agents, *Prunus*

## Abstract

*Prunus mahaleb* has garnered attention as a potent medicinal agent and functional component. We aimed to detect the chemical composition and biological activities of several parts (fruit, leaves, and twigs) of *P. mahaleb* . Biological activities were assessed for antioxidant properties, enzyme inhibition, mutagenic/antimutagenic effects, and antibacterial efficacy. Antioxidant capabilities were evaluated using various assays, including DPPH, ABTS, CUPRAC, FRAP, phosphomolybdenum, and metal chelating. The chemical constituents of the extracts were identified and quantified using the HPLC‐ESI‐MS/MS method. The effects of enzyme inhibition were examined on some enzymes, including AChE, BChE, tyrosinase, amylase, and glucosidase. The Ames test was used to evaluate the mutagenic and antimutagenic properties of the plant extracts. Furthermore, a broth microdilution assay was employed to evaluate the possible antibacterial effects of the extracts against microorganisms. The methanol extract of twigs showed superior antioxidant capabilities (DPPH: 388.39 mg TE/g; ABTS: 701.50 mg TE/g; CUPRAC: 459.05 mg TE/g; FRAP: 264.99 mg TE/g). The methanol extract of twigs demonstrated the highest tyrosinase inhibitory activity (61.91 mg KAE/g). A total of 40 metabolites, mainly flavonoids, were detected through HPLC‐ESI‐MS/MS analysis, revealing that ferulic acid, naringenin, and herniarin were the predominant compounds. In the Ames test, the tested extracts exhibited no mutagenic potential. The antimutagenicity assay demonstrated that methanol and ethyl acetate extracts from twigs and leaves were particularly efficient against frameshift and base pair substitution mutations induced by recognized mutagens. The metabolic activation system amplified these strong activities to inhibition rates ranging from 85% to 98%. The results from the antibacterial assay indicated antibacterial effectiveness at dosages between 6.25 and 0.195 mg/mL, particularly effective against *Sarcina lutea, Bacillus cereus, Candida albicans*, and *Staphylococcus aureus* . Our findings indicate that *P. mahaleb* can serve as a versatile raw material for the development of health‐promoting applications, including medicines, cosmeceuticals, and nutraceuticals.

## Graphical abstract

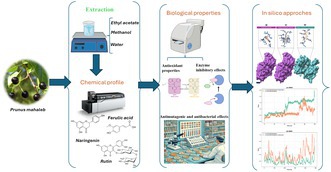

## 1 Introduction

For centuries, plants have been utilized by humans, and although they can be used in various ways such as for food, in textile industries, and building shelters (Mostafa et al. 2016; Fathallah et al. 2023), their role as herbal medicine remains one of the earliest forms of health care. The study of plants dates back hundreds of years, allowing for their use as food and for the extraction of non‐food products (Aly et al. 2021). From a scientific perspective, a medicinal plant contains pharmacologically active compounds (Mostafa et al. 2018), which enable it to be used, either directly or indirectly, in treatments to prevent or heal specific ailments. Numerous studies have aimed at identifying the medicinal properties of plants, including their potential anti‐inflammatory, anti‐cancer, anti‐diabetic, and antioxidant effects (Veiga et al. 2020). Furthermore, many widely used modern medicines are derived from plants, and their therapeutic effects are believed to stem from the various compounds found in these plants, such as terpenoids, alkaloids, flavonoids, phenolics, and others. According to the World Health Organization, over 80% of the global population depends on traditional plant‐based medicine for their primary healthcare (Veiga et al. 2020; WHO 2019). Even today, plants remain essential in healthcare and represent a promising source for safe future medicines. Despite the availability of many modern drugs, there is still a pressing need to discover and develop new therapeutic agents. It is estimated that effective treatments are only available for about one‐third of the known human diseases. As a result, the battle against illnesses must continue without pause. Traditional plant‐based medicines still hold a significant place in today's pharmaceutical industry, owing to their minimal side effects and the synergistic effects of the combination of compounds (Aly et al. 2021, 2022; Hamburger and Hostettmann 1991; Dar et al. 2017).

The genus *Prunus*, which belongs to the Rosaceae family and the Prune subfamily, comprises around 430 species. These species are distributed across five subgenera: *Padus, Amygdalis, Cerasus, Prunophora*, and *Laurocerasus*. They include deciduous and evergreen trees and shrubs that predominantly grow in the temperate regions of the Northern Hemisphere. most of these species are of significant horticultural value. Many of them are cultivated for their edible seeds and fruits, such as *P. amygdalis* (almonds), *P. domestica* (plums), *P. persica* (peaches), *P. cerasus* (cherries), and *P. americana* (apricots). Additionally, a large number of these species are highly regarded for their ornamental value due to their attractive flowers. The wood of several *Prunus* species is used for various purposes. For instance, the wood of *P. avium* is utilized in the production of high‐quality furniture, the wood of *P. puddum* is used for crafting walking sticks and umbrella handles, while the heartwood of *P. domestica* is used in cabinet making, inlay work, and turning, and *P. amygdalus* heartwood is valued in turnery and marquetry (Poonam et al. 2011). Several of these species are used for their medicinal properties. The leaves and flowers of *P. spinosa* are known for their lithontriptic and diuretic effects and are included in the diets of individuals suffering from peptic ulcers. The stems of *P. avium* and *P. cerasus* are beneficial in treating certain heart conditions. The fruit of *P. salicina* is used in the treatment of arthritis. An aqueous extract made from the small branches of *P. cerasoides* is taken internally to prevent abortion. An infusion made from the leaves and bark of this plant is used for conditions such as whooping cough, asthma, dyspepsia, and diarrhea. Additionally, the leaves and flowers of *P. cerasoides* are employed in treating kidney stones and gravel disease. This plant is believed to aid digestion, so it is included in the diet for treating ulcers. Its heartwood is also useful in addressing pitta imbalances (such as burning sensations), sprains, and skin discoloration (Poonam et al. 2011).

Mahaleb ( *Prunus mahaleb* L. belongs to the Rosaceae family) is a member of the genus *Prunus*. *mahaleb* is a short shrub (sometimes up to 10–15 m tall) deciduous tree with scattered and broad crowns, fruit and branches with a special scent, and white flowers (Hedberg and Staugard 1989; Mariod et al. 2010) Although the native land of maize is Europe and Western Asia, it naturally spreads over a wide area that extends to Southern Europe, France, Southern Germany, Northern Asia, the Caucasus, and the depths of Turkistan (Farag et al. 2021). The species is thermophilic, drought‐resistant, and requires abundant summer heat. It thrives well on skeletal and chalky dry soils, and sometimes even grows in meadow areas (Clinovschi 2005). The parts of the plant are used as a traditional medicine in Turkish medicine for the treatment of diabetes, gastrointestinal problems, and various ailments (Seyyednejad et al. 2008). Additionally, the resin obtained from the outer surface of the wood is used in the treatment of gastritis. The oil extracted from the seeds is used in the production of liqueurs and special wines due to its aromatic taste. The fruits are used in pastries and bakeries (Özçelik et al. 2012). In Sudan, crushed white mahaleb kernels are used in the production of traditional fragrances. Additionally, the consumption of soaked white mahaleb seeds is considered a remedy for the treatment of diarrhea in children (Mariod et al. 2009).

A comprehensive review of the extant literature revealed the presence of as yet unstudied properties in the *P. mahaleb* plant. The present study therefore sought to evaluate this plant using a variety of in vitro and in silico methods, including anti‐mutagenicity, antimicrobial, antioxidant, enzyme inhibition, molecular docking, as well as a detailed content analysis by HPLC.

## 2 Material and Methods

### 2.1 Plant Materials and Extraction Procedures

Different parts and fruit samples of *P. mahaleb* , which will be evaluated within the study, were collected mainly during summer at Alaeddin Keykubat Campus of Selçuk University. The first step was to identify the specimens based on their morphological characteristics systematically. The systematic identification of the plant was carried out by Assoc. Prof. Dr. Bayram ATASAGUN (Selcuk University, Vocational School of Health Services). Some of the identified samples have been deposited and the specimen number assigned (B. Atasagun‐1104).

The maceration and infusion methods were used to extract the samples. For this purpose, ethyl acetate, methanol, and water were chosen as solvents to extract the branches, leaves, and fruit parts of the plant. To prepare maceration extracts, plant samples (5 g) were mixed with 100 mL of solvent (methanol, ethyl acetate) for 24 h at room temperature (in the dark) using a shaking incubator. The infusion method was used for the aqueous extract. 5 g of plant sample was added to boiled water at 100°C and kept for 15 min. The extracts were filtered and the solvents evaporated. The extracts obtained at the end of the study were stored in dry form at +4°C (Zengin et al. 2020).

### 2.2 UPLC‐ESI‐MS Analysis

A comprehensive analysis was carried out using HPLC‐ESI‐MS/MS as mentioned by (Aly et al. 2023) to examine the chemical composition of *P. mahaleb* . The samples were introduced into a Shimadzu 8045 UPLC system (Kyoto, Japan), coupled with a triple quadrupole mass analyzer from Shimadzu Corporation. The extracts were diluted in HPLC‐grade methanol and filtered through a 0.2 μm polytetrafluoroethylene (PTFE) filter. Chromatographic separation of compounds was performed using a Shimpack C18 reversed‐phase column with a particle size of 2.7 μm and dimensions of 2 × 150 mm. Gradient elution was conducted with solvents A (water) and B (acetonitrile) at a flow rate of 0.2 mL/min. The elution began with 10% B for 5 min, then a gradual increase to 30% B over 15 min, and then to 70% B within 22 min. The concentration was further raised to 80% B from 22 to 30 min before being reduced back to 10% B at 35 min.

Negative electrospray ionization (ESI) was used for mass measurement. The temperature at the contact was set to 300°C, and the temperature at the desolvation point stayed at 526°C. It was set so that 50 L/h of cone gas would flow and 3 L/min of nebulizing gas would flow. We performed collision‐induced dissociation (CID) in MS/MS mode by changing the impact energy for each peak separately, between 20 and 50 eV. The mass range that was analyzed by mass spectrometry was from 100 to 1200 m/z. The Lab Solutions program was used to process data. Thermo Scientific in Karlsruhe, Germany, made the XcaliburTM 2.0.7 program that was used to collect and analyze the data.

#### 2.2.1 Assay for Total Phenolic and Flavonoid Contents

Total phenolics and flavonoids were quantified in our previous paper (Slinkard and Singleton 1977). Folin–Ciocalteu and AlCl_3_ methods were used to determine the total phenolic and flavonoid contents, respectively. Gallic acid (GA) and rutin (R) served as reference standards in the experiments, with results reported as gallic acid equivalents (GAE) and rutin equivalents (RE).

#### 2.2.2 Assays for In Vitro Antioxidant Capacity

As previously described (Grochowski et al. 2017), various antioxidant tests were performed. DPPH, ABTS radical scavenging, CUPRAC, and FRAP results were milligrams of Trolox equivalents (TE) per gram. In millimoles of TE per gram of extract, the phosphomolybdenum (PBD) test measured antioxidant potential, and in EDTA, metal chelating activity (MCA) was measured.

#### 2.2.3 Inhibitory Effects Against Some Key Enzymes

In accordance with the established protocols (Grochowski et al. 2017), experiments on enzyme inhibition were performed on the samples. Acarbose equivalents (ACAE) per gram of extract were used to measure the activities that inhibit amylase and glucosidase, while milligrams of galanthamine equivalents (GALAE) per gram of extract were used to examine the inhibition of acetylcholinesterase (AChE) and butyrylcholinesterase (BChE). The amount of tyrosinase inhibition for each gram of extract was measured in milligrams of kojic acid equivalents (KAE).

### 2.3 Ames Test

*P. mahaleb* extracts were subjected to an (Anti) mutagenicity test for their potential genotoxic/antigenotoxic activities. The plaque incorporation method described by Maron and Ames (1983) was performed with some modification through *Salmonella Typhimurium* TA98 and TA100 strains (Nibras Qader Qader et al. 2022). The inhibition rates obtained from the test were evaluated according to Negi et al. (2003).

### 2.4 Antimicrobial Activity

All of the strains used in our studies were obtained from the Microbiology Research Laboratory of Selçuk University, Vocational School of Health Services. The broth microdilution test was used to evaluate the antimicrobial properties of *P. mahaleb* extracts. The test was performed according to Koc and Uysal (2016) with some modifications.

### 2.5 Studies on Pharmacokinetics and ADME (Absorption, Distribution, Metabolism, and Excretion)

To discover whether the main classes of compounds could be promising drugs, the Absorption, Distribution, Metabolism, and Excretion (ADME) and Pharmacokinetic Studies were conducted using SWISSadme (Swiss Institute of Bioinformatics online source), link: www.swissadme.ch, accessed on 2 July 2024. Lipinski's rule of five was assessed to evaluate the drug‐likeness of the compounds (Chen et al. 2020; Alomar et al. 2025). The physicochemical properties for oral bioavailability were detected by the Swiss ADME molecules' bioavailability radar. The pink area represents the ideal spaces for six physicochemical assets, such as polarity, size, solubility, lipophilicity, flexibility, and saturation, for the oral bioavailability of the representative compound. The boiled egg approach was also utilized for predicting the blood‐ brain barrier and GIT absorption of the compound (Fathallah et al. 2023; Yousuf et al. 2022).

### 2.6 Protein and Ligand Preparation

Molecular docking analysis was performed to investigate the interactions of the major phytochemicals in *P. mahaleb* extract. All 3D structures of selected proteins, identified using the Pharos platform (https://pharos.nih.gov/), were downloaded from the Protein Data Bank (PDB) (https://www.rcsb.org/) by searching with their respective PDB IDs. AChE (PDB ID: 2Y2V) (Cetiz et al. 2024a), BChE (PDB ID: 3DJY) (Yagi et al. 2024a), amylase (PDB ID: 2QV4) (Korpayev et al. 2025), glucosidase (PDB ID: 3W37) (Cetiz et al. 2024b), and tyrosinase (PDB ID: 5M8O) (Cusumano et al. 2024). Furthermore, molecular docking was conducted for antimicrobial‐related proteins, including the 30S ribosome ( *S. aureus* (PDB ID: 5TCU) and *E. coli* (PDB ID: 4V53)), dihydropteroate synthase ( *S. aureus* (PDB ID: 1AD4) and *E. coli* (PDB ID: 5V7A)), gyrase B ( *S. aureus* (PDB ID: 4URN) and *E. coli* (PDB ID: 1KZN)), muramyl ligase E (MurE) ( *S. aureus* (PDB ID: 4C13) and *E. coli* (PDB ID: 1E8C)), transpeptidase ( *S. aureus* (PDB ID: 5TW8) and *E. coli* (PDB ID: 6NTW)), and B‐DNA dodecamer (PDB ID: 1BNA) (Cetiz et al. 2024a; Korpayev et al. 2025; Saqallah et al. 2022). All ligands and proteins were prepared for molecular docking using Avogadro V1.2.0, BIOVIA Discovery Studio, and AutoDock V4.2.6 (Trott and Olson 2009).

### 2.7 Docking Grid and Parameters

The docking grid files were generated based on the literature or using POCASA V1.1 (https://g6altair.sci.hokudai.ac.jp/g6/service/pocasa/) (Duran et al. 2024; Yu et al. 2010). AChE (X: 31.062, Y: 20.311, Z: 11.947; 22 Å × 30 Å × 40 Å), BChE (X: 44.794, Y: −19.63, Z: −25.227; 30 Å × 30 Å × 30 Å), tyrosinase (X: −13.194, Y: 5.341, Z: −26.28; 26 Å × 26 Å × 28 Å), amylase (X: 14.188, Y: 48.964, Z: 22.886; 28 Å × 28 Å × 24 Å), and glucosidase (X: 3.091, Y: −8.008, Z: −4.08; 42 Å × 52 Å × 54 Å). Additionally, molecular docking was performed for antimicrobial targets, including 30S ribosome S3 ( *S. aureus* , PDB ID: 5TCU, coordinates: X: 99.46, Y: 230.082, Z: 201.387; grid box size: 76 Å × 76 Å × 76 Å) and ( *E. coli* , PDB ID: 4 V53, coordinates: X: 130.966, Y: 32.099, Z: 0.385; grid box size: 88 Å × 88 Å × 88 Å), dihydropteroate synthase ( *S. aureus* , PDB ID: 1 AD4, coordinates: X: 32.46, Y: 6.683, Z: 42.972; grid box size: 60 Å × 60 Å × 60 Å) and ( *E. coli* , PDB ID: 5V7A, coordinates: X: −17.836, Y: −17.836, Z: 103.740; grid box size: 60 Å × 60 Å × 60 Å), gyrase B ( *S. aureus* , PDB ID: 4URN, coordinates: X: −31.684, Y: −5.252, Z: 1.572; grid box size: 60 Å × 60 Å × 40 Å) and ( *E. coli* , PDB ID: 1KZN, coordinates: X: 12.467, Y: 27.336, Z: 44.916; grid box size: 60 Å × 60 Å × 60 Å), muramyl ligase E (MurE) ( *S. aureus* , PDB ID: 4C13, coordinates: X: −23.122, Y: 2.508, Z: 9.873; grid box size: 60 Å × 60 Å × 60 Å) and ( *E. coli* , PDB ID: 1E8C, coordinates: X: 45.098, Y: 37.112, Z: 76.674; grid box size: 70 Å × 60 Å × 70 Å), transpeptidase ( *S. aureus* , PDB ID: 5TW8, coordinates: X: 21.390, Y: −62.210, Z: 39.196; grid box size: 60 Å × 60 Å × 60 Å) and ( *E. coli* , PDB ID: 6NTW, coordinates: X: 16.929, Y: −32.370, Z: 42.151; grid box size: 60 Å × 60 Å × 60 Å).

### 2.8 Validation and Interaction Analysis

The grid box dimensions were specified according to the respective protein‐ligand binding sites. AutoDock Vina V1.1.2 (https://autodock.scripts.edu) (Morris et al. 2009) was used to identify distinct ligand conformations, with the exhaustiveness parameter set to 32 (Trott and Olson 2009). To validate the docking accuracy, proteins were re‐docked with their co‐crystallized ligands, and Root Mean Square Deviation (RMSD) values were computed (Akpulat et al. 2025). To obtain more profound insight into protein‐enzyme‐ligand interactions, researchers employed the Protein‐Ligand Interaction Profiler (PLIP) (https://plip‐tool.biotec.tu‐dresden.de/plip‐web/plip/index), which highlighted critical interactions, particularly hydrogen bonds (Angeles Flores et al. 2024; Llorent‐Martínez et al. 2025). These analytical methods confirmed that our docking results were precise and reliable.

### 2.9 Molecular Dynamic Simulation

Molecular dynamics simulations were conducted using the CHARMM‐GUI platform (https://charmm‐gui.org), and the system was prepared with the Solution Builder tool (Jo, Kim, Iyer, & Im, 2008). The CHARMM36m force field was used to parameterize proteins, establishing a periodic boundary box with TIP3P water molecules while ensuring a minimum separation of 10 Å from the box edges (Maier et al. 2015; Yagi et al. 2024b). To achieve electroneutrality, counterions were introduced to regulate the NaCl concentration at 0.15 M, contributing to system stability. To enhance the accuracy of the simulation, electrostatic and van der Waals interactions were managed via the Verlet cutoff scheme, whereas bond constraints were applied through the linear constraint solver (LINCS) algorithm. Additionally, long‐range electrostatic interactions were computed using the particle mesh Ewald (PME) method (Angeles Flores et al. 2024). To ensure system stability, energy minimization was performed via the steepest descent algorithm, thereby reducing potential energy fluctuations to levels below 1000 kJ/mol/nm (Korpayev et al. 2025). Following energy minimization, equilibration was performed under NPT and NVT conditions at 310 K to ensure thermodynamic stability. Thereafter, production simulations were executed using GROMACS 2023.3 for a total duration of 100 ns (Kurt‐Celep et al. 2025).

### 2.10 Calculation of MM/PBSA Free Energy to Determine Ligand‐Binding Affinity

Molecular dynamics (MD) simulations were performed for a duration of 50–250 frames to evaluate the stability and binding affinity of various protein‐ligand complexes. The gmx_MMPBSA (Mechanics/Poisson‐Boltzmann Surface Area) V1.6.3 (https://valdes‐tresanco‐ms.github.io/gmx_MMPBSA/dev/getting‐started/) was utilized to calculate the free binding energy and assess the stability of each system. The protein‐ligand complexes subjected to 10 ns MD simulations included *S. aureus* ‐MurE_Quercetin, *S. aureus* ‐MurE_Naringenin, *S. aureus* ‐MurE_Kaempferol‐7‐O‐glucoside, *E. coli* ‐Transpeptidase_Kaempferol‐7‐O‐glucoside, *S. aureus* ‐Transpeptidase_Kaempferol‐7‐O‐glucoside, *E. coli* ‐30S ribosome S3_Kaempferol‐7‐O‐glucoside, and *E. coli* ‐MurE_Kaempferol‐7‐O‐glucoside (Miller III et al. 2012; Valdés‐Tresanco et al. 2021).

### 2.11 Statistical Analysis

The experiments were repeated three times, and extract differences were evaluated using One‐way ANOVA with Tukey's post hoc test. Statistical analysis was carried out in GraphPad Prism (version 9.2), considering p‐values below 0.05 as statistically significant.

## 3 Results and Discussion

### 3.1 Total Phenolic and Flavonoid Contents

Phenolic compounds are versatile active ingredients in nutraceutical and pharmaceutical applications. In particular, they are considered effective antioxidants and therefore protect against the attack of free radicals. In this sense, the determination of the total phenol content can be carried out as a first finding. In this study, the total content of phenolics and flavonoids was determined using colorimetric methods. Obviously, the content of these bioactive compounds depends on the extraction solvents used. The highest total phenol levels were found in the methanol extract of twigs with 107.93 mg GAE/g, followed by water (88.14 mg GAE/g) and ethyl acetate (75.64 mg GAE/g) of the twigs (Table 1). The lowest total phenol content was found in the water extract of fruits (8.79 mg GAE/g). As for the total flavonoids, the ethyl acetate and methanol extracts contained more flavonoids in each part than water. The highest total flavonoid content was 36.37 mg RE/g in the ethyl acetate extract from twigs.

**TABLE 1:** The total phenolic and flavonoid contents in *P. mahaleb* extracts.

Parts	Solvents	Total phenolic content (mg GAE/g)	Total flavonoid content (mg RE/g)
Leaves	Ethyl acetate	10.53 ± 0.18^fg^	16.31 ± 0.45^c^
	Methanol	17.36 ± 1.26^e^	27.23 ± 0.65^b^
	Water	17.18 ± 0.08^e^	10.87 ± 0.32^d^
Fruits	Ethyl acetate	21.81 ± 0.73^d^	2.70 ± 0.32^e^
	Methanol	12.12 ± 0.22^f^	1.55 ± 0.06^f^
	Water	8.79 ± 0.22^g^	1.01 ± 0.02^g^
Twigs	Ethyl acetate	75.64 ± 0.80^c^	36.67 ± 0.93^a^
	Methanol	107.93 ± 1.09^a^	27.18 ± 1.35^b^
	Water	88.14 ± 0.72^b^	10.12 ± 0.09^d^

*Note:* Values are reported as mean ± SD of three parallel experiments. Different letters indicate significant differences between the tested extracts (*p* < 0.05).

Abbreviations: GAE: Gallic acid equivalents; RE: Rutin equivalents.

Different levels of the total number of bioactive compounds in *P. mahaleb* extracts have been reported in the literature. Similar to our observations, Taghizadeh et al. (2015) found that the highest level of the total phenolic content was in the bark extracts and the lowest content in the fruit extracts. In addition, Pehlivan (2021) detected the highest level of the total phenolic content in bark extract, as compared to leaf and fruit. The total content of phenol and flavonoids in the seed extract of *P. mahaleb* was reported by Younis et al. (2024) as 34.37 mg GAE/100 g and 85.48 mg QE/100 g. In another study by Gercek et al. (2023), the total content of phenol and flavonoids in the methanol extract of *P. mahaleb* fruits was 5.49 mg GAE/100 g fresh weight and 3.08 mg QE/100 g fresh weight. The differences can be explained by geographical and climatic factors. In addition, colorimetric methods for these bioactive compounds do not fully reflect the actual levels of these compounds. In particular, not only phenolic but also other compounds can react with the reagents, and the results obtained may be incorrect (Nikolaeva et al. 2022). In this regard, the spectrophotometric results need to be confirmed by chromatographic methods such as HPLC‐MS and NMR.

### 3.2 UPLC/MS

The chemical profiling of the leaves, fruits, and stems of *P. mahaleb* was done by using UPLC‐ESI‐QTOF‐MS in both positive and negative ionization modes. The Metabolites were eluted according to their polarity in descending order (40 metabolites) and then tentatively identified conferring their molecular ion peaks, MS/MS data, and after comparing with the literature (Table 2). The spectral interpretation revealed a total of 27, 19, and 15 compounds in the leaves, fruits, and stems, respectively. The stems' ethyl acetate, methanol, and aqueous extracts showed 10, 8, and 8 compounds, respectively, while the fruits showed 3, 3, and 7 compounds. The leaves displayed 7, 5, and 9 compounds. The main metabolites were divided into four categories (i.e., flavonoids, coumarins, anthocyanins, and hydro‐cinnamic derivatives) based on their structural characteristics.

**TABLE 2:** Key metabolites detected in the ethyl acetate (EtOAc), methanol, and water extracts of *P. mahaleb* from Turkey assisted by HPLC‐ESI‐MS/MS analysis arranged according to their molecular weights where (E) stands for 10 *10^.

Peak no.	*t*_R_	[M‐H]^−^	[M + H]^+^	MS^2^	Tentatively identified compounds	Phytochemical class	Intensity									Ref.
							*P. mahaleb* Leaves			*P. mahaleb* fruits			*P. mahaleb* stems			
							EtOAc	Methanol	Water	EtOAc	Methanol	Water	EtOAc	Methanol	Water	
Phenolic acids																
1	1.27	115.0115	—	115	Fumaric acid	Phenolic acid	—	—	—	—	—	2.8E4	—	—	—	Zan et al. (2022); Kenneth and Carlson (1975)
2	0.79	—	149.0225	149, 95	Cinnamic acid	Unsaturated carboxylic acid	—	—	—	—	—	—	1.6E6	—	—	Bayrakçeken Güven et al. (2023)
3	1.63	—	181.0465	181, 109	Caffeic acid	Hydroxycinnamic acid	1.3E6	—	—	—	—	—	—	—	—	Kenneth and Carlson (1975)
4	31.36	167.0401	—	167.0401	Vanillic acid	Phenolic acid	—	—	—	—	—	1.3E4	—	—	—	Orlando et al. (2021)
5	1.08	191.0562	—	191, 133	Quinic acid	Phenolic acid	—	—	—	—	—	—	—	2.1E5	—	Kenneth and Carlson (1975)
6	6.00	193.0560	195.0528	193, 149	Ferulic acid	Phenolic acid (hydroxycinnamic acid derivative)	—	—	3.1E6	—	—	6.3E5	—	—	—	Özcan et al. (2023)
7	5.56	195.0579	—	195, 81	Di‐hydro ferulic acid	Dihydro‐phenolic acid	—	—	—	—	—	—	1.2E6	—	—	Mostafa and Farag (2023)
8	10.37	341.0934	—	341, 264, 181	Caffeic acid 3‐glucoside	Phenolic acid glucoside	—	—	—	—	4.1E6	—	—	—	4.4E4	Zan et al. (2022)
9	19.69	—	355.0998	355.193	Chlorogenic acid	Phenolic acid	—	—	—	—	—	1.7E6	—	—	—	Ibrahim et al. (2023)
10	6.33	357.1180	—	357, 194, 134	2‐(Glucosyloxy)‐4‐methoxy‐cinnamic acid	Cinnamic acid derivative glycoside	—	—	—	—	2.5E6	1.4E7	—	—	—	El‐Dakhakhny (1970)
11	6.83	710.2125	—	710,531, 324, 271, 193	Ferulic acid‐o‐hexoside dimer	Hydroxycinnamic acid glycoside derivative	—	1.6E5	7.3E5	—	—	—	—	—	—	Mostafa and Farag (2023)
12	5.44	715.2332	—	715, 357, 195	Dihydroferulic acid‐O‐ hexoside dimer	Hydroxycinnamic acid glycoside derivative	—	—	—	—	—	—	—	3.4E6	—	Mostafa and Farag (2023)
Flavonoids																
13	11.69	269.0953	—	269, 194, 111	Apigenin	Flavonoid	—	—	—	—	—	—	—	—	3.4E4	Ghafoor et al. (2019)
14	8.75	271.0616	—	271, 243, 175, 130	Naringenin	Flavonoid	—	—	—	—	—	—	1.3E7	4.7E6	9.6E5	Mikulic‐Petkovsek et al. (2016); Zhukovets and Özcan (2021)
15	5.88	285.0541	—	285, 229, 135	Kaempferol	Flavonol	—	—	—	—	—	—	4.2E5	3.8E5	—	Mikulic‐Petkovsek et al. (2016)
16	9.73	287.0483	—	287, 227, 194	Eriodictyol	Flavanone	—	1.7E5						2.4E7	—	Mostafa and Farag (2023)
17	9.48	289.0646	291.0810	291, 114	Catechin	Flavan‐3‐ol	2.9E5	—	—	—	—	—	—	—	—	Ghafoor et al. (2019) Orlando et al. (2021)
18	9.48	289.0646	291.0810	291, 114	Epi‐catechin	Flavan‐3‐ol	2.9E5	—	—	—	—	—	—	—	—	Ghafoor et al. (2019) Orlando et al. (2021)
19	7.02	301.0460	—	301, 180, 70, 50	Quercetin	Flavonol	—	—	—	—	—	—	7.6E5	9.0E4	—	Mikulic‐Petkovsek et al. (2016)
20	1.20	315.0610	—	315, 265, 230, 180, 163, 132, 114	Isorhamnetin	O‐methylated flavon‐ol	—	—	1.8E5	4.6E5	—	—	—	—	—	Gercek et al. (2023); Zhukovets and Özcan (2021)
21	7.38	431.1088	—	431, 271, 169, 125	Afzelin	Flavonol glycoside	—	6.8E4	3.7E4	1.6E6	—	—	—	—	—	Zan et al. (2022)
22	5.86	447.0955	449.0649	447, 226, 165	Quercitrin	Flavonol glycoside	—	—	3.7E5	—	—	—	—	—	—	Zan et al. (2022)
23	5.78	447.0955	449.0649	447, 354, 302, 285, 193, 116	Kaempferol 7‐O‐glucoside	Flavonol glycoside	—	—	7.0E6	—	—	2.4E5	9.1E5	—	—	Zan et al. (2022)
24	7.05	463.0822	465.0985	463, 324, 287, 160	Hyperoside	Flavonol glycoside							6.5E5	1.6E6	1.1E5	Gercek et al. (2023)
25	8.71	609.1591	—	609, 447, 309	Rutin	Flavonoid glycoside	—	5.6E4	—	—	—	—	—	—	—	Gercek et al. (2023)
26	3.66	623.1655		623, 327, 289, 116	Narcissin	Flavonol glycoside	—	—	—	—	—	—	—	—	4.4E4	Gercek et al. (2023)
27	5.96	711.1284	—	711, 355, 193	Quercetin 3‐O‐(6″‐malonyl‐glucoside) 7‐O‐glucoside	Flavonol glycoside	—	—	7.9E6	—	—	—	—	—	—	Popović et al. (2021)
28	5.49	755.1999	—	755, 354, 297, 180	Quercetin 3‐O‐rhamnosyl‐(1‐>2)‐rhamnosyl‐(1‐>6)‐glucoside	Flavonol Glycoside	—	—	2.6E4	1.2E5	—	—	—	—	—	Zan et al. (2022)
Coumarins																
29	2.59	—	147.0208	147	Coumarin	Coumarin	—	—	—	—	—	9.8E5	—	—	—	Jerković et al. (2011)
30	8.02	175.0335	177.0498	177, 129	Herniarin	Coumarin	—	2.0E6	—	—	—	—	—	—	1.4E7	Mostafa and Farag (2023)
31	11.44	—	327.1231	327,291, 175, 109	*Trans*‐o‐Coumaric acid 2‐glucoside	Coumaric acid glycoside	3.0E5	—	—	—	—	—	—	—	—	Blando et al. (2016)
32	2.11	—	465.1509	465, 304, 163,113	Taxifolin 7‐glucoside	Coumarin Glycoside	—	—	—	—	—	—	3.7E5	—	—	Zan et al. (2022)
Anthocyanins																
33	2.51	577.1496	—	577, 465, 289	Procyanidin B1	Procyanidin dimer.	—	—	—	—	—	—	5.8E5	—	—	Mikulic‐Petkovsek et al. (2016)
34	12.23	610.1673	—	470, 378, 244	Cyanidin 3,5‐diglucoside	Anthocyanin glycoside	—	—	—	—	—	—	—	—	4.4E4	Ieri et al. (2012)
35	6.83	615.1001	—	615, 417, 369, 194	Cyanidin 3‐sambubioside	Anthocyanin glycoside	1.4E5	—	—	—	—	—	—	—	—	Ieri et al. (2012)
36	12.23	772.1940	—	772, 652, 470, 378, 244	Cyanidin 3,5‐diglucoside dimer	Anthocyanidin glycoside	—	—	—	—	—	—	—	—	4.4E4	Ieri et al. (2012)
Miscellaneous																
37	5.75	151.0302	153.0375	153, 110, 60	Vanillin	Phenolic aldehyde	4.5E5	—	—	—	—	—	7.1E6	—	—	Mostafa and Farag (2023)
38	6.51	165.0598	—	165, 100.931	Caffeoyl alcohol	Catechol derivative	1.4E5	—	—	—	—	—	—	—	—	Farag et al. (2021)
39	21.28	—	283.2595	282,239, 140,102	Oleic acid	Fatty acid	—	—	6.7E5	—	4.2E5	—	—	—	—	Ibrahim et al. (2023)
40	2.39	289.0287	—	289, 161, 112	Unknown	—	—	—	—	—	—	—	—	6.4E5	—	

Regarding the phenolic acids (hydroxycinnamic acid derivatives) and their glycosides, it was noted that they were distributed among all the extracts and were observed in both positive and negative ionization modes with relatively more abundance in the negative mode. Not only were 12 metabolites identified according to their molecular weights, but also their characteristic MS^2^ as described by (Ostrowski et al. 2014). The fruit aqueous extract exhibited the highest number of phenolic acids, as 5 metabolites were marked in it, namely: Fumaric acid, Vanillic acid, ferulic acid, 2‐(Glucosyloxy)‐4‐methoxy‐cinnamic acid, and caffeic acid glycoside with their deprotonated molecular ion at m/z 115, 151, 193, 357, 149, and 341 respectively. Ferulic acid was the most dominant phenolic acid along with its glycosides and dimers. It appeared in both ionization modes and in the three different extracts of the plant. The deprotonated ion peak was m/z 193 in negative mode and m/z 195 in positive mode. The MS^2^ revealed a fragment of m/z 149 due to the loss of CHO_2_ from the side chain.

Not only does the presence of phenolic acids in plants aid them in varied functions, from nutrient uptake to photosynthesis, but they also enhance the physical, nutritional, and antioxidant capacities of edible ones (Robbins 2003). The wide distribution of those compounds in *P. mahaleb* 's organs (leaves, fruits, and stems) plays an important role in the antioxidant and protective effect of the plant.

Flavonoids and their glycosides were the major metabolites found in the various plant extracts with more abundance in the negative ionization compared to the positive ionization mode. It was noted that the stems and the leaves contained most of the aglycone flavonoids and their glycosides, namely: apigenin, Naringenin, kaempferol, catechin, epicatechin, quercetin, quercitrin, hyperoside, rutin, narcissin, and Quercetin 3‐O‐(6″‐malonyl‐glucoside) 7‐O‐glucoside. Nevertheless, the fruit extracts were nearly devoid of the free (aglycone) flavonoids yet had some methylated and flavonoid glycosides such as isorhamnetin, Afzelin, kaempferol‐7‐glucoside, and Quercetin 3‐O‐rhamnosyl‐(1‐>2)‐rhamnosyl‐(1‐>6)‐glucoside. The MS^2^ of the flavonoids was typical of that described by (Cuyckens and Claeys 2004). Naringenin was the most abundant flavonoid metabolite with a deprotonated ion peak of m/z 271 and fragments of m/z 243 due to loss of CO, C_12_H_15_O m/z 175, then the opening of the C ring with m/z 130.

As reported in previous research papers (Bose et al. 2018; Shahrajabian et al. 2022; Bekhouche et al. 2022), flavonoids are present in many plants, fruits, vegetables, and leaves which may have uses in medicine. Among its many medical advantages are flavonoids' antiviral, cardio‐ and neuroprotective properties, antioxidant, anticancer, and anti‐inflammatory merits. The presence and dominance of those metabolites in *P. mahaleb* may be partially responsible for its biological activities, as illustrated by (Pehlivan 2021).

Coumarin, Herniarin, Trans‐o‐Coumaric acid, and 2‐glucoside Taxifolin 7‐glucoside were spotted mainly in the positive ionization mode with molecular ion peaks of 147, 177, 327, and 465, respectively. The mass fragmentation displayed a typical pattern of coumarin derivatives with the loss of CO_2_ followed by the opening of the ring and the loss of C_2_H_2_ as reported by (Concannon et al. 2000). Those coumarins were previously isolated and identified as the major coumarins found in *P. mahaleb* by (Mostafa and Farag 2023; Jerković et al. 2011). Most of the coumarins were found in the leaf extracts such as herniarin, trans‐o‐Coumaric acid, and taxifolin 7‐glucoside, while coumarin was present in the fruit extract. The use of fruit and seeds as flavoring agents in the baking industry, especially in cookies (Herken et al. 2017) may be related to coumarin, as previously described by (Al‐Said and Hifnawy 1986; Ieri et al. 2012) as it is well known to have an aroma similar to vanilla; however, herniarin is a well‐established coumarin with its ability to reduce inflammation and protect neurons (Liliana Porras‐Dávila et al. 2023).

Anthocyanidins were observed exclusively in the negative ionization mode. Procyanidin B1, Cyanidin 3,5‐diglucoside dimer, and Cyanidin 3,5‐diglucoside were the anthocyanin glycosides found in the stem extracts. Cyanidin 3‐sambubioside was detected in the EtoAc of the leaves. Notably, those metabolites were completely absent from the fruits. The MS^2^ appeared to follow the patterns described by (Giusti et al. 1999). An eminent example, procyanidin B, revealed a deprotonated ion peak of m/z 577, llowed by m/z 465.17 due to the successive loss of the hydroxyl groups, and m/z 289 from the breakage of the dimer bonding.

Other miscellaneous classes of active metabolites were noticed in the three extracts. Oleic acid appeared in the positive mode of the fruits' EtoAc and aqueous extracts with protonated ion peaks at m/z 283 and MS^2^ peaks at m/z140 and m/z 102 due to the loss of (C_8_H_15_O_2_•) and (C_13_H_25_•) from the aliphatic chain, respectively. Caffeoyl alcohol and vanillin are phenolic compounds and were detected in the leaves and stems extracts with no trace in the fruits' extracts.

The variation in the chemical profiling of the three extracts might be responsible for the several pharmacological uses of the plant; besides, it was proven from this study that the choice of the extraction solvent may be a crucial factor in obtaining the desired active constituents, as mentioned before by (Boeing et al. 2014).

### 3.3 Antioxidant Properties

Antioxidants have a great interest in protecting against free radical attacks. Therefore, they can alleviate serious health problems such as cancer, cardiovascular disease, and diabetes. The antioxidant mechanisms are based on various effects, and this one universal method is not possible to measure the antioxidant potential of a plant extract. In the current study, the antioxidant abilities of the tested extracts were investigated through various chemical tests (Sadeer et al. 2020). The results are shown in Table 3. DPPH and ABTS are the most common tests and were used to evaluate the radical‐scavenging effect of plant extracts. In both tests, the methanol and water extracts of the individual parts were more active than the ethyl acetate extracts. The strongest free radical scavenger was found in the methanol extract from branches with 388.39 mg TE/g (in DPPH) and 701.50 mg TE/g (in ABTS). Reducing ability is another important mechanism for evaluating antioxidant capacity and reflects the ability of antioxidants to donate electrons. CUPRAC and FRAP assays were carried out for this purpose. Similar to the DPPH and ABTS assays, the best reduction abilities were found in the branch extracts. They decreased in the following order: methanol > water > ethyl acetate. In general, fruit extracts had the weakest abilities (24.35–27.72 mg TE/g in CUPRAC; 13.84–17.99 mg TE/g in FRAP). The results of radical scavenging and the reducing capacity are almost identical to the total phenol content. In line with our findings, several researchers reported a linear correlation between total phenol content and antioxidant properties (Zhang et al. 2025; Belew and Gebre 2025; Esmaeili et al. 2025). The phosphomolybdenum test also involves a reduction reaction, namely the conversion of Mo (VI) to Mo (V). As can be seen from Table 3, the highest ability was found in the phosphomolybdenum test in the ethyl acetate extract of leaves with 2.23 mmol TE/g. In general, the fruit extracts were weaker than the leaf and branch extracts in the phosphomolybdenum test. The metal chelate formation is linked to the binding of transition metals in the Fenton reaction and thus controls the production of hydroxyl radicals. In each part, the water extracts showed a stronger metal‐chelating effect than the extracts of ethyl acetate and methanol. The best ability was found in the aqueous leaf extract with 25.04 mg EDTAE/g, while the ethyl acetate extract from fruits has the weakest metal chelating capacity (1.45 mg EDTAE/g).

**TABLE 3:** Antioxidant properties of the extracts from *P. mahaleb* .

Parts	Solvents	DPPH (mg TE/g)	ABTS (mg TE/g)	CUPRAC (mg TE/g)	FRAP (mg TE/g)	PBD (mmol TE/g)	MCA (EDTAE/g)
Leaves	Ethyl acetate	4.30 ± 0.60^ef^	15.06 ± 0.62^g^	36.60 ± 0.48^e^	15.04 ± 0.25^e^	2.23 ± 0.02^a^	23.11 ± 1.02^b^
	Methanol	15.75 ± 0.26^d^	60.46 ± 2.05^d^	47.78 ± 1.49^d^	23.54 ± 1.85^d^	1.71 ± 0.14^b^	23.46 ± 0.24^b^
	Water	16.71 ± 0.98^d^	61.52 ± 1.71^d^	27.20 ± 0.73^f^	21.76 ± 0.57^de^	0.39 ± 0.01^f^	25.04 ± 0.13^a^
Fruits	Ethyl acetate	0.69 ± 0.04^f^	2.71 ± 0.34^h^	27.72 ± 0.27^ef^	13.84 ± 1.10^e^	0.99 ± 0.03^cd^	1.45 ± 0.17^e^
	Methanol	12.65 ± 0.61^de^	43.11 ± 2.96^e^	26.16 ± 0.44^f^	17.99 ± 0.20^de^	0.79 ± 0.13^de^	11.74 ± 0.60^d^
	Water	8.44 ± 1.02^def^	32.23 ± 0.66^f^	24.35 ± 0.88^f^	15.57 ± 0.56^e^	0.58 ± 0.04^ef^	23.93 ± 0.27^ab^
Twigs	Ethyl acetate	209.40 ± 1.94^c^	300.71 ± 3.82^c^	260.31 ± 3.55^c^	163.35 ± 1.44^c^	1.43 ± 0.24^b^	17.32 ± 0.50^c^
	Methanol	388.39 ± 8.14^a^	701.50 ± 3.17^a^	459.05 ± 7.55^a^	264.99 ± 7.82^a^	1.47 ± 0.03^b^	16.05 ± 0.53^c^
	Water	257.64 ± 3.95^b^	440.84 ± 4.00^b^	335.61 ± 3.90^b^	214.97 ± 1.09^b^	1.13 ± 0.01^c^	22.61 ± 0.11^b^

*Note:* Values are reported as mean ± SD of three parallel experiments. Different letters indicate significant differences between the tested extracts (*p* < 0.05).

Abbreviations: EDTAE, EDTA equivalents; MCA, metal chelating; PBD, phosphomolybdenum; TE, trolox equivalents.

The literature review highlighted that the antioxidant properties of *P. mahaleb* extracts or fractions have been documented by authors from various countries. For example, Guven et al. (Güven et al. 2023) reported that the antioxidant properties of seeds and kernels of *P. mahaleb* from Turkey and the kernel extract had a stronger ABTS scavenging ability than the seed extract. In another study by Gercek et al. (Gercek et al. 2023), the abilities of the methanol extract of *P. mahaleb* in CUPRAC and ABTS assays were 3.92 mg TE/100 g and 1.11 mg TE/100 g, respectively. The stem extracts of *P. mahaleb* exhibited higher DPPH radical scavenging ability compared to leaf and fruit, as reported by Taghizadeh et al. (2015). Pehlivan (2021) reported that the fruit (90.2%) and bark extracts (88.23%) exhibited stronger DPPH radical scavenging than leaf extract (79.35%). As an insight into structure–ability, the observed results in the current study can be attributed to the chemical components of the tested extracts. Table 2 shows that ferulic acid, naringenin, quercetin, and hyperoside were the main ingredients and are known as effective antioxidants (Cavia‐Saiz et al. 2010; Zduńska et al. 2018; Qi et al. 2022; Jang 2022).

### 3.4 Enzyme Inhibitory Effects

Day by day, the prevalence of some diseases has been rising over the past decade, influenced by changes in lifestyle. For example, 537 million adults are living with diabetes in 2021, and the number is expected to be 643 million in 2030 (Ogurtsova et al. 2022). There is therefore an urgent need to act against this problem. To this end, enzymes play the main role in alleviating these diseases. The inhibition of specific enzymes can mitigate the symptoms of the disease, leading to the selection of key targets such as cholinesterase for Alzheimer's, amylase for diabetes, and tyrosinase for skin diseases. In pharmacy, several compounds have been used as enzyme inhibitors to achieve this goal. However, most of them show unpleasant side effects. We therefore need alternative, safe, and powerful inhibitors to replace synthetic ones (Patil et al. 2022). Based on this background, we investigated the inhibitory effects of the tested extracts. The results are summarized in Table 4. Similar to antioxidant activities, the enzyme‐inhibiting effects depended on the extraction solvents used. The best AChE and BChE inhibitory actions were observed in ethyl acetate extract from leaves (AChE: 4.06 mg GALAE/g; BChE: 3.80 mg GALAE/g). In each part, the ethyl acetate extract was more active in AChE than in methanol and water extracts. However, the water extract of leaves was not effective for AChE and BChE. Tyrosinase inhibition is related to the control of hyperpigmentation problems. As shown in Table 4, the strongest tyrosinase inhibition was found in methanol extract from twigs (61.91 mg KAE/g), followed by ethyl acetate extracts from twigs (46.44 mg KAE/g) and leaves (45.77 mg KAE/g).

**TABLE 4:** Enzyme inhibitory properties of the extracts from *P. mahaleb*.

Parts	Solvents	AChE (mg GALAE/g)	BChE (mg GALAE/g)	Tyrosinase (mg KAE/g)	Amylase (mmol ACAE/g)	Glucosidase (mmol ACAE/g)
Leaves	Ethyl acetate	4.06 ± 0.19^a^	3.80 ± 0.10^a^	45.77 ± 1.80^b^	0.34 ± 0.03^b^	0.83 ± 0.04^d^
	Methanol	2.82 ± 0.35^c^	2.35 ± 0.25^b^	36.78 ± 0.50^cd^	0.22 ± 0.01^c^	0.12 ± 0.03^e^
	Water	na	na	na	0.05 ± 0.01^e^	0.06 ± 0.01^f^
Fruits	Ethyl acetate	3.45 ± 0.19^b^	2.19 ± 0.01^b^	33.32 ± 2.86^d^	0.43 ± 0.01^a^	2.28 ± 0.01^b^
	Methanol	3.16 ± 0.01^bc^	2.19 ± 0.20^b^	37.56 ± 0.27^c^	0.21 ± 0.01^cd^	2.07 ± 0.11^c^
	Water	2.86 ± 0.10^c^	2.39 ± 0.27^b^	36.52 ± 1.14^cd^	0.17 ± 0.01^d^	2.14 ± 0.01^c^
Twigs	Ethyl acetate	3.49 ± 0.05^b^	1.45 ± 0.04^c^	46.44 ± 1.19^b^	0.35 ± 0.02^b^	2.43 ± 0.01^a^
	Methanol	na	1.22 ± 0.16^c^	61.91 ± 0.53^a^	0.37 ± 0.01^b^	2.41 ± 0.03^a^
	Water	0.81 ± 0.02^d^	0.35 ± 0.05^d^	27.76 ± 1.12^e^	0.06 ± 0.01^e^	2.11 ± 0.01^c^

*Note:* Values are reported as mean ± SD of three parallel experiments. Different letters indicate significant differences between the tested extracts (*p* < 0.05).

Abbreviations: ACAE, acarbose equivalents; GALAE, galatamin equivalents; KAE, kojik acid equivalents; na, not active.

The inhibition of amylase and glucosidase is associated with a delay in the rise of blood sugar levels in diabetics. In the amylase inhibition assay, the ethyl acetate and methanol extracts were more active than the water extracts for each part. The best amylase inhibition was found in the ethyl acetate fruit extract with 0.43 mmol ACAE/g. As far as glucosidase inhibition is concerned, the strongest ability was found in twig extracts, and the methanol and water extracts showed almost the same effects. In the literature, several researchers reported enzyme inhibitory properties of *P. mahaleb* extracts. For example, Orlando et al. (Orlando et al. 2021) investigated the enzyme inhibitory effect of the water extract of the fruit of *P. mahaleb* , and the extract exhibited strong AChE, BChE, tyrosinase, and glucosidase inhibition with a low IC_50_ value (< 2 mg/mL). In another study by Güven et al. (2023), the ethyl acetate extracts of shells, seeds, and fruits of *P. mahaleb* exhibited significant tyrosinase inhibition potentials at 100 μg/mL (41.19%–60.44%). In addition, some compounds were isolated from the kernels of *P. mahaleb* , and 2‐O‐β‐glucopyranosyloxy‐4‐methoxy‐hydrocinnamic acid exhibited a great tyrosinase inhibition with an IC50 value of 0.22 mM. The amylase and glucosidase inhibitory potential (IC_50_) of the cherry of P. mahaleb was reported by Popović et al. (2021) as 43.95 and 0.96 mg/mL. In the current study, the observed enzyme‐inhibiting effects can be attributed to the presence of some ingredients, including ferulic acid (Mugundhan et al. 2024; Alifah et al. 2024; Zheng et al. 2020), naringenin (Tran et al. 2021; Li et al. 2022, 2014), quercetin (Huang et al. 2024; Liao et al. 2022; Silva et al. 2023), hyperoside (Wang et al. 2023; Shen et al. 2023; Li et al. 2019) etc. in the extracts tested. Taken together, *P. mahaleb* can be considered a source of natural enzyme inhibitors in the development of functional ingredients.

### 3.5 Mutagenicity and Antimutagenicity

The results showing the number of revertant colonies and their standard deviations of the mutagenicity test performed with the plate incorporation method are given in Table 5.

**TABLE 5:** Mutagenicity (Ames) test results of *Prunus mahaleb* extracts on *S. typhimurium* TA 98 and TA 100 strains in the presence and absence of S9.

	Concentration (μg/plate)	TA98		TA100	
		S9 (−)	S9 (+)	S9 (−)	S9 (+)
Positive^a^ control		742 ± 23	3226 ± 156	2511 ± 160	5138 ± 190
Negative^b^ control	100 μL/plate	35 ± 4	35 ± 3	109 ± 5	154 ± 11
Bacteria control		31 ± 3	36 ± 4	141 ± 9	142 ± 11
*Twig water*	5000	31 ± 1	39 ± 1	123 ± 2	148 ± 10
	2500	33 ± 4	33 ± 3	132 ± 4	151 ± 8
	1000	29 ± 3	32 ± 2	135 ± 3	142 ± 8
*Twig methanol*	10,000	31 ± 2	42 ± 4	156 ± 9	153 ± 9
	5000	33 ± 3	41 ± 2	162 ± 8	150 ± 10
	2500	32 ± 2	35 ± 0	155 ± 7	161 ± 7
*Twig ethyl acetate*	10,000	35 ± 1	32 ± 4	152 ± 5	154 ± 7
	5000	31 ± 4	34 ± 2	159 ± 1	152 ± 9
	2500	29 ± 0	30 ± 2	151 ± 3	142 ± 5
*Leaf water*	5000	29 ± 1	41 ± 0	126 ± 4	152 ± 6
	2500	37 ± 1	38 ± 2	144 ± 6	143 ± 3
	1000	32 ± 3	37 ± 1	151 ± 4	148 ± 5
*Leaf methanol*	10,000	33 ± 4	30 ± 3	137 ± 5	151 ± 6
	5000	30 ± 1	33 ± 7	140 ± 6	145 ± 7
	2500	29 ± 3	30 ± 1	141 ± 8	152 ± 9
*Leaf ethyl acetate*	10,000	31 ± 3	29 ± 4	135 ± 4	141 ± 6
	5000	33 ± 3	33 ± 3	142 ± 8	145 ± 7
	2500	29 ± 4	40 ± 2	135 ± 1	154 ± 4
*Fruit water*	10,000	30 ± 3	29 ± 3	145 ± 3	146 ± 7
	5000	31 ± 0	35 ± 1	135 ± 2	143 ± 10
	2500	34 ± 3	34 ± 2	138 ± 4	139 ± 8
*Fruit methanol*	10,000	31 ± 2	36 ± 2	140 ± 3	158 ± 10
	5000	27 ± 3	38 ± 2	150 ± 3	147 ± 5
	2500	30 ± 3	29 ± 3	138 ± 7	139 ± 8
*Fruit ethyl acetate*	10,000	24 ± 0	32 ± 1	143 ± 3	141 ± 5
	5000	29 ± 3	36 ± 2	133 ± 5	161 ± 4
	2500	30 ± 4	34 ± 3	154 ± 4	148 ± 6

^a^ *Positive control:* 2‐Aminofluorene (7.5 μg/plate) was used as a positive indirect mutagen in the presence of S9 mixture; 4‐nitro‐O‐phenylenediamine (5 μg/plate) was used as a positive direct mutagen for *S. typhimurium* strain TA98 without S9 mixture. 2‐Aminoanthracene (5 μg/plate) was used as a positive indirect mutagen in the presence of S9 mixture; Sodium azide (5 μg/plate) was used as a positive direct mutagen without S9 mixture for *S. typhimurium* TA100.

^b^ *Negative control:* DMSO (100 μL/plate) was used as a negative control for *S. typhimurium* TA98 and TA100 both in the presence and absence of S9.

Water, methanol, and ethyl acetate extracts of twigs, leaves, and fruit parts of the *P. mahaleb* plant were subjected to a mutagenicity test at non‐toxic doses. Experiments were performed both in the presence and absence of metabolic activation. When the findings were compared with bacterial control numbers, the results showed that all of the extracts did not cause more than twice the number of bacteria compared to the control plate. In other words, all of the extracts do not have a mutagenic effect at non‐toxic doses. This situation was the same in the presence of the S9 mixture, meaning that they did not have a secondary mutagenic potential (Table 5).

The lack of mutagenic potential of the plant extracts tested in the study brought to mind the question of whether they have any antimutagenic properties against known mutagens, and in this context, the inhibition rates of the extracts were studied both in the presence and absence of S9. Table 6 shows the number of revertant colonies and inhibition percentages of the extracts against mutagens.

**TABLE 6:** Antimutagenicity results and percentage inhibition of *P. mahaleb* extracts against direct and indirect mutagens determined on *S. typhimurium* TA98 and TA100 strains in the presence and absence of metabolic activation (S9).

	Concentration (μg/plate)	TA 98				TA 100			
		S9 (−)	% inhibition	S9 (+)	% inhibition	S9 (−)	% inhibition	S9 (+)	% inhibition
Positive^a^ control		742 ± 23	0	3226 ± 156	0	2511 ± 160	0	5138 ± 190	0
Negative^b^ control	100 μL/plate	35 ± 4		35 ± 3		109 ± 5		154 ± 11	
Bacteria control		31 ± 3		36 ± 4		141 ± 9		142 ± 11	
*Twig water*	5000	458 ± 30	40	1682 ± 176	48	2491 ± 11	1	4096 ± 111	21
	2500	493 ± 13	35	2960 ± 59	8	2369 ± 98	6	4849 ± 131	6
	1000	500 ± 13	34	3006 ± 214	7	2785 ± 3	0	4120 ± 160	20
*Twig methanol*	10,000	322 ± 12	59	202 ± 1	95	1577 ± 118	39	246 ± 6	98
	5000	426 ± 22	44	326 ± 16	91	1906 ± 194	26	302 ± 23	97
	2500	478 ± 61	37	440 ± 16	87	2174 ± 94	14	341 ± 0	96
*Twig ethyl acetate*	10,000	322 ± 8	59	268 ± 8	93	2024 ± 137	21	364 ± 22	96
	5000	377 ± 10	51	224 ± 8	94	2177 ± 107	14	309 ± 16	97
	2500	406 ± 0	47	264 ± 35	93	2044 ± 36	20	363 ± 18	96
*Leaf water*	5000	416 ± 37	46	2572 ± 214	21	2873 ± 146	0	5729 ± 203	0
	2500	448 ± 11	41	2875 ± 182	11	2627 ± 28	0	6061 ± 197	0
	1000	546 ± 39	27	3048 ± 88	6	2627 ± 83	0	5144 ± 151	0
*Leaf methanol*	10,000	445 ± 45	42	238 ± 30	94	2405 ± 112	4	417 ± 30	94
	5000	455 ± 35	40	529 ± 47	85	2527 ± 5	0	1063 ± 90	82
	2500	481 ± 36	37	1956 ± 8	40	2607 ± 54	0	2213 ± 181	59
*Leaf ethyl acetate*	10,000	452 ± 24	41	78 ± 11	99	1274 ± 48	52	298 ± 42	97
	5000	493 ± 1	35	162 ± 23	96	1552 ± 11	40	396 ± 32	95
	2500	523 ± 24	31	231 ± 21	94	1651 ± 36	36	632 ± 86	90
*Fruit water*	10,000	411 ± 14	46	2350 ± 117	27	2207 ± 51	13	6338 ± 159	0
	5000	494 ± 27	35	2235 ± 134	31	2184 ± 39	14	5781 ± 189	0
	2500	480 ± 34	37	2116 ± 113	35	2459 ± 103	2	5662 ± 214	0
*Fruit methanol*	10,000	479 ± 13	37	2807 ± 81	13	1712 ± 100	34	3229 ± 97	38
	5000	501 ± 28	34	1995 ± 1	39	2489 ± 32	1	4945 ± 107	4
	2500	568 ± 14	24	2582 ± 132	20	2506 ± 116	0	4813 ± 152	7
*Fruit ethyl acetate*	10,000	520 ± 4	31	1744 ± 135	46	2696 ± 118	0	3756 ± 167	28
	5000	505 ± 83	33	2312 ± 141	29	2822 ± 80	0	5287 ± 148	0
	2500	526 ± 11	30	2620 ± 174	19	2789 ± 70	0	5079 ± 194	1

^a^ Positive control: 2‐Aminofluorene (7.5 μg/plate) was used as a positive indirect mutagen in the presence of S9 mixture; 4‐nitro‐O‐phenylenediamine (5 μg/plate) was used as a positive direct mutagen for *S. typhimurium* strain TA98 without S9 mixture. 2‐Aminoanthracene (5 μg/plate) was used as a positive indirect mutagen in the presence of S9 mixture; Sodium azide (5 μg/plate) was used as a positive direct mutagen without S9 mixture for *S. typhimurium* TA100.

^b^ Negative control: DMSO (100 μL/plate) was used as negative control for *S. typhimurium* TA98 and TA100 both in the presence and absence of S9.

According to the findings obtained in the study conducted with the Salmonella/microsome test, *P. mahaleb* twig water extract improved the mutagenic effect of 4‐NPDA in the absence of S9 for the TA98 strain with a strong inhibition of 40% at the highest concentration of 5000 μg/plate. At the remaining doses of 2500 and 1000 μg/plate, it revealed moderate antimutagenic activity with inhibition of 35% and 34%, respectively. Twig water extract exhibited a strong antimutagenic effect against 2‐AF at a dose of 5000 μg/plate with the addition of metabolic activation enzymes to the test medium (Table 6). In the remaining doses, the addition of S9 enzyme was reported to be weakly effective against 2‐AF, reducing the moderate effect. When this extract was evaluated for the TA100 strain, it did not show any antimutagenic effect ameliorating the base pair substitution mutation against SA in the absence of S9 and against 2‐AA in the presence of S9 (Table 6). Twig methanol extract showed a strong antimutagenic effect against 4‐NPDA at doses of 10,000 and 5000 μg/plate for the TA98 strain in the absence of S9, with inhibition rates of 59% and 44%, respectively. In the presence of metabolic activation enzymes, they very strongly reversed the frameshift mutation caused by 2‐AF with inhibition rates of 95%, 91%, and 87% at all doses, respectively, and demonstrated a very strong antimutagenicity. Twig methanol extract caused a moderate antimutagenicity with inhibitions of 39% and 26% against SA at concentrations of 10,000 and 5000 μg/plate in the absence of S9 for strain TA100. After the addition of S9 mixture to the test medium, it showed near‐perfect inhibition rates (98%, 97%, and 96%) against 2‐AA at all test doses. In this case, it can be said that the addition of metabolic activation strongly reversed the base pair exchange mutation. *P. mahaleb* twig ethyl acetate extracts were able to strongly combat frameshift mutation at doses of 10,000, 5000, and 2500 μg/plate with inhibition rates of 59%, 51%, and 47% for strain TA98 in the absence of S9. After the addition of S9 enzymes, these ratios showed improvement against 2‐AF with a remarkably strong inhibition of 93%, 94%, and 93%, respectively. While twig ethyl acetate extract showed weak antimutagenic effect in the absence of S9 for the TA100 strain, interestingly, after the addition of metabolic activation enzymes, it showed near‐perfect antimutagenicity with a marvelous increase of 96%, 97%, and 96% inhibition rates at all test doses. As a general evaluation, it can be said that except for twig water extract, methanol and ethyl acetate extracts strongly increase their activities with the addition of S9.

Leaf water extract exhibited a strong antimutagenic effect against frameshift mutation, with rates of 46% and 41% at doses of 5000 and 2500 μg/plate for strain TA98. On the contrary, this extract lost its activity and did not show any effect against the mutagen 2‐AF in the presence of S9. A similar situation was recorded against the mutagenic effects of SA and 2‐AA, both in the presence and absence of S9 for the TA100 strain. There were no inhibitory effects against these mutagens. It is thought that this situation indicates these activities are due to different contents in the leaf and twig extracts. While the leaf methanol extract showed a strong antimutagenic effect against 4‐NPDA at doses of 10,000 and 5000 μg/plate for the TA98 strain, the inhibition rates shown to 2‐AF with the addition of S9 were evaluated as 94%, 85%, and 40%, respectively, indicating strong antimutagenicity. For the TA100 strain, while the methanol extract could not show any effect in the absence of S9, it was able to fight against the base pair exchange mutation effect caused by 2‐AA with very strong rates (94%, 82%, 59% respectively) with the addition of metabolic activation (Table 2).

Ethyl acetate extracts of the leaf showed strong to moderate antimutagenicity for strain TA98 in the absence of S9, while the addition of the enzyme improved the effect of 2‐AF with near‐perfect inhibition rates of 99%, 96%, and 94%. A similar situation was observed in strain TA100 while it showed strong activity of 52% and 40% at 10000 and 5000 μg/plate doses in an S9‐free medium, it was reported that metabolic activation induced high‐level activities of 97%, 95%, and 90% for all test doses, respectively.

Mahaleb fruit water extract was found to be strongly antimutagenic against 4‐NPDA at a dose of 10,000 μg/plate for strain TA98 and showed moderate activity at other doses. After the addition of S9, all doses were found to be moderately antimutagenic (27%, 31%, 35%, respectively). For strain TA100, fruit water extract exhibited no antimutagenicity against mutagens both in the presence and absence of S9. Fruit methanol extract showed a moderate effect at 10,000 and 5,000 μg/plate doses for TA98 in the absence of S9 (37%, 34%), while this did not change after the addition of S9 at some doses and decreased at others. Strain TA100 was able to fight against SA and 2‐AF with moderate antimutagenic activity only at 10000 μg/plate in the presence and absence of metabolic enzymes (Table 6). When the fruit ethyl acetate extract was evaluated, moderate antimutagenicity was shown against the mutagenic effect of 4‐NPDA at all test doses for strain TA98. With the addition of S9 enzyme, an increase in activity was observed only at the highest dose and an inhibition rate of 46% against 2‐AF was detected. While no inhibition rate could be determined in the absence of enzymes for strain TA100, moderate activity against 2‐AA was shown with S9 addition only at 10000 μg/plate dose with 28% (Table 6).

In recent years, studies on *P. mahaleb* have attracted attention. In the antimutagenicity study of fruit extracts conducted with *Saccharomyces cerevisiae* yeast, it was reported that no cytotoxic and mutagenic effects were observed at doses of 0.1–3 mg/mL. It was reported that 25‐fold antimutagenicity was observed against mitotic gene conversion compared to control, and three‐fold antimutagenicity was observed in reversing point mutations compared to control plate. In vitro tests on yeast cells have shown that concentrated mahaleb fruit extract has a protective effect resulting from its ability to lower intracellular ROS levels due to its strong free radical scavenging activity (Gerardi et al. 2016). In another study, the anticancer and mutagenic and antimutagenic potentials of copper nanoparticles (CuNP) synthesized using *P. mahaleb* extracts were evaluated by Ames test. The results showed that the synthesized nanoparticles had high cytotoxicity, but no mutagenic effect and exhibited over 40% antimutagenicity at all test doses. It was emphasized that this was due to nanoparticles (Dashtizadeh et al. 2021). In the present study, no mutagenic effects were found in the twig, leaf, and fruit extracts of *P. mahaleb* (Table 6). However, it was reported that especially methanol and ethyl acetate extracts of leaves and twigs, in the presence of metabolic activation enzymes, reversed base pair exchange and frameshift mutations at a high rate and showed very strong antimutagenicity. In this context, our results are in agreement with the results of other researchers.

The phytochemical substances as flavonoids and the other phenolic compounds are presented in nutrients and herbal medicines; both flavonoids and many other phenolic components have been reported on their effective antioxidants, anticancer, antibacterial, cardioprotective agents, anti‐inflammation, immune system promoting, skin protection from UV radiation, and interesting candidates for pharmaceutical and medical applications (Tungmunnithum et al. 2018). In our study, the major phenolic compounds revealed by HPLC analysis were ferulic acid and vanillin; also, flavonoid derivatives were Naringenin, quercetin, kaempferol 7‐O‐glucoside, and hyperoside. So, coumarin was detected for the coumarin phytochemical class. It was reported that naringenin has anticancer, antimutagenic, anti‐inflammatory, antioxidant, antiproliferative, and antiatherogenic activities. Naringenin is used for the treatments of osteoporosis, cancer, and cardiovascular diseases, and showed lipid‐lowering and insulin‐like properties (Patel et al. 2018). In a study conducted by Birosova et al. (2005), the phenolic acids such as caffeic acid, gallic acid, and ferulic acid had an antimutagenic capacity against 3‐(5‐nitro‐2‐furyl)acrylic acid (5‐NFAA) and sodium azide. The authors manifested that ferulic acid was the second effective compound against mutagens. The antimutagenicity of vanillin was screened by Ames test against the mutagenic effects of well‐known mutagens, and these significant antimutagenic capacities were reported by some researchers (Yoshida et al. 2015; Moreno et al. 2023). Quercetin was reported for its antimutagenic potential against oxidative mutagen t‐butylhydroperoxide, and with the addition of ascorbic acid, the increase in antimutagenicity was observed (Geetha et al. 2005). In another study, diethylnitrosamine‐induced DNA damage and apoptosis were ameliorated by quercetin in rats (Gupta et al. 2010) and also other detailed pharmacological properties were reported by Carrillo‐Martinez et al. (2024). Kaempferol has been found to have anti‐mutagenic as well as genotoxic properties (Ross and Kasum 2002). Antigenotoxic activity of hyperoside was defined by Yuzbasioglu et al. (2023) against Mitomycin C and H_2_O_2_. When the results of the researchers and the results obtained from this study were compared, it was seen that the results were quite compatible. In the present study, it was established that the incorporation of metabolic activation enzymes served to augment the antimutagenic properties of the extracts. This phenomenon can be explained as follows: The antimutagenic effect is triggered by the competitive inhibition of P450 isoenzymes by liver glycosides (Edenharder et al. 1993). Previous studies have demonstrated that certain plant metabolites are potent inhibitors of cytochrome P450 reductase (Buening et al. 1981). Kappus (1986) demonstrated that the preventive activity following metabolic activation is associated with the role of cytochrome P450 isoforms in detoxification systems, either through reductase or oxygenase functions. These functions serve as antioxidants, scavenging compounds that neutralize free radicals, oxygen radicals, and reactive oxygen species (Parke et al. 1991). Consequently, the most significant antimutagenic activity observed in assays in the presence of a metabolic fraction may be associated with the activation of a cytochrome P450 that facilitates the oxidation of promutagens (Mitscher et al. 1996), suggesting that this effect could be a consequence of the competitive inhibition by glycosides of cytochrome P450, thereby preventing the formation of the promutagen (Edenharder et al. 1993; Akin et al. 2016). The significant biological activities of *P. mahaleb* extracts can be attributed to phenolics and flavonoids.

### 3.6 Antimicrobial Activity

Water, methanol, and ethyl acetate extracts of twigs, leaves, and fruit parts of *P. mahaleb* were evaluated for their antimicrobial potential. The minimum inhibition concentrations (MIC) determined by the broth microdilution test are given in Table 7.

**TABLE 7:** Antimicrobial activity results of extracts of *P. mahaleb* plant.

MIC values (mg/mL)										
Strains	1	2	3	4	5	6	7	8	9	Gentamicin (μg/mL)
*Escherichia coli* ATCC 25922	—	—	—	—	—	1.56	—	—	—	1.95
*Pseudomonas aeruginosa* ATCC 27853	—	—	—	—	—	1.56	—	—	—	< 0.97
*Klebsiella pneumoniae* ATCC 700603	—	—	—	—	—	—	—	—	—	7.81
*Staphylococcus aureus* (MRSA)ATCC 43300	6.25	—	1.56	—	3.12	0.781	6.25	3.25	—	1.95
*Salmonella enteritidis* ATTC 13076	—	1.56	—	—	—	—	3.125	1.56	—	1.95
*Sarcina lutea* ATCC 9341	6.25	0.781	1.56	—	—	0.195	—	—	—	1.95
*Bacillus cereus* ATTC 11778	3.125	0.781	0.781	6.25	0.781	1.56	—	—	—	1.95
*Candida albicans* NRRL Y‐417	6.25	1.56	0.781	—	1.56	0.781	6.25	—	—	7.81

*Note:* 1, twig water; 2, twig methanol; 3, twig ethyl acetate; 4, leaf water; 5, leaf methanol; 6, leaf ethyl acetate; 7, fruit water; 8, fruit methanol; 9, fruit ethyl acetate.

According to the results obtained from the study, the twig water extract was found to be effective only against gram‐positive bacteria *S. aureus* , *S. lutea* , and *B. cereus* , and the MIC values determined for these strains were 6.25, 6.25, and 3.125 mg/mL, respectively. It was also antifungal against *C. albicans* at a dose of 6.25 mg/mL. All of the remaining strains were found to be resistant to the twig water extract. When twig methanol extract was evaluated, it was found to be effective against *S. enteritidis* , a Gram‐negative pathogenic bacterium, at a dose of 1.56 mg/mL, and showed antibacterial activity against *S. lutea* and *B. cereus* at a concentration of 0.781 mg/mL. It was recorded to be antifungal at a dose of 1.56 mg/mL for *Candida*. Methicillin‐resistant *S. aureus* , *E. coli* , *P. aeruginosa* , and *K. pneumoniae* were not affected by this extract. Twig ethyl acetate extract was effective against gram‐positive bacteria at doses ranging from 0.781 to 1.56 mg/mL. The MIC values were 1.56 mg/mL for MRSA and *S. lutea* , and 0.781 mg/mL for *B. cereus* and *C. albicans* (Table 3).

The water extract of *P. mahaleb* leaves was ineffective for all strains except *B. cereus* . The MIC value determined for *B. cereus* was 6.25 mg/mL. Leaf methanol extract was effective against *B. cereus* at a dose of 0.781 mg/mL, while it showed antimicrobial activity against methicillin‐resistant *S. aureus* at a dose of 3.12 mg/mL and *C. albicans* at a dose of 1.56 mg/mL. In general, the leaf ethyl acetate extract was effective against the remaining microorganisms except *K. pneumoniae* and *S. enteritidis* among the strains tested. Only this extract was effective against *E. coli* and *P. aeruginosa* at doses of 1.56 mg/mL. It was also able to fight against *S. aureus* and *C. albicans* at a dose of 0.781 mg/mL. The MIC value determined for *B. cereus* was 1.56 mg/mL. The lowest MIC value throughout the study was observed against *S. lutea* at a dose of 0.195 mg/mL.

The fruit water extract revealed antimicrobial potential against *S. aureus* and *C. albicans* at a dose of 6.25 mg/mL and against *S. enteritidis* at a dose of 3.125 mg/mL. Similarly, only *S. aureus* and *S. enteritidis* were affected by the fruit methanol extract, and the MIC values determined against these strains were reported as 3.25 and 1.56 mg/mL, respectively. Fruit ethyl acetate extract, on the other hand, showed no effect against any of the tested microorganisms, and all strains tested were resistant to this extract.

When the whole study was evaluated, the lowest MIC value was determined against *S. lutea* at a dose of 0.195 mg/mL of leaf ethyl acetate extract. Leaf ethyl acetate extract was the most effective extract at various doses (1.56–0.195 mg/mL) against the remaining strains except *K. pneumoniae* and *S. enteritidis* . It was also the only extract effective against *E. coli* and *P. aeruginosa* . *K. pneumoniae* was the most resistant bacterium against the extracts tested, while fruit ethyl acetate extract was the least effective extract. Another noteworthy point in the study is that the extracts were more effective against Gram‐positive bacteria than Gram‐negative bacteria. Dashtizadeh et al. (2021) evaluated the antimicrobial activity of copper nanoparticles obtained from *P. mahaleb* extracts. The researchers reported that the nanoparticles synthesized from the extract were most effective on Gram‐positive bacteria. Ozcelik et al. (2012) reported that methanol and hexane extracts obtained from *P. mahaleb* twig, leaf, flower, seed kernel, and fresh fruit parts had antibacterial effects on Gram‐positive bacteria at doses of 16–64 μg/mL, antibacterial effects on Gram‐negative bacteria at doses of 16–64 μg/mL, and antifungal effects on *C. krusei* species at a dose of 64 μg/mL. A previous study conducted with ethanol extracts of *P. mahaleb* seeds using the disc diffusion method to determine antimicrobial activity against various gram‐negative and gram‐positive bacterial species showed that *P. mirabilis* was the most sensitive bacterium at a dose of 0.4 g/mL. In addition, an inhibitory effect was also observed against Gram‐positive bacteria *B. anthracis* and *S. aureus* at the same concentration (Ozcelik et al. 2012). Eliuz et al. (2022) reported that *P. mahaleb* seed oil has antifungal effect on *C. albicans* and *C. parasilopsis* at a dose range of 102.5–17.9 μg/mL. In the present study, water, methanol, and ethyl acetate extracts (except fruit ethyl acetate) of *P. mahaleb* twig, leaf, and fruit parts were found to have antibacterial effect on Gram‐positive bacteria and Gram‐negative bacteria at doses ranging from 6.25 to 0.195 mg/mL. In addition, except for leaf water, fruit methanol, and ethyl acetate extracts, the remaining extracts showed antifungal effect on *C. albicans* at doses between 6.25 and 0.781 mg/mL. In this context, the results of the above‐mentioned researchers and the results of the present study were found to be compatible with each other in terms of antibacterial and antifungal activity.

### 3.7 Swiss ADME Prediction Online Software (Lipinski's Rule of Five, Radar Plot, and Boiled Egg Method)

To predict the possible expediency of the identified compounds as real future drugs before proceeding to the In vivo studies, a pharmacokinetic study had to be conducted by measuring many parameters such as Absorption, Distribution, Metabolism, and Excretion (ADME) using SwissADME (Daina et al. 2017; Salah et al. 2024; Fathallah et al. 2024), which is an online tool created by the Swiss Institute of Bioinformatics, accessed on July 2, 2024. Firstly, the Lipinski Rule of Five, which helps determine whether possible compounds are suitable for biological systems, was used to evaluate the physicochemical characteristics of those compounds that were relevant to oral bioavailability (Fathallah et al. 2023; Attique et al. 2019). Secondly, The SwissADME bioavailability radar plot feature establishes the optimal limits for six important properties: size, polarity, solubility, lipophilicity, flexibility, and saturation. Besides, the Brain Or Intestinal EstimateD permeation approach (BOILED‐egg) strategy was applied by the software program as a predictive measure for the polarity and lipophilicity assessment of the tested compounds. This allowed for the prediction of two crucial pharmacokinetic parameters: the compound's ability to cross the blood–brain barrier and its absorption in the gastrointestinal tract (GIT).

In this study, most of the compounds were found to obey Lipinski's conditions to be oral drugs. Nevertheless, some compounds were found to violate the rule in one or more parameters. As seen in Table 8 all the phenolic acids and the coumarins, obeyed the requirements of the rule except for taxifolin 7‐glucoside which violated two parameters in the number of hydrogen donors and acceptors. Despite that all the aglycone (free) flavonols' obeyed the rule; it was noted that violations of up to three parameters were recorded in 5 of the glycosides, namely Quercitrin, kaempferol 7‐O‐glucoside, Hyperoside, Rutin, Narcissin, Quercetin 3‐O‐(6″‐malonyl‐glucoside) 7‐O‐glucoside, and Quercetin 3‐O‐rhamnosyl‐(1‐>2)‐rhamnosyl‐(1‐>6)‐glucoside. The violations included molecular weights of more than 500 g/mol, the number of hydrogen donors being more than 5 atoms, and the hydrogen acceptors being more than 10 atoms. Regarding the anthocyanins glycosides, all of them were violating the rule as their molecular weights were bigger than 500 g/mol, and their number of hydrogen atoms acceptors and donors was more than 10 and 5, respectively.

**TABLE 8:** Lipinski's rule of five for the main classes identified from the *P. mahaleb* plant.

Name	M.wt	Hydrogen Bond acceptors	Hydrogen Bond Donors	Lipophilicity	No. of rule violations	Drug‐likeness
	Less than 500 g/mol	Less than 10	Less than 5	Less than 5	Less than 2 violations	Lipinski's rule follows rule
Phenolic acids						
Fumaric acid	116.07 g/mol	4	2	−0.64	0	Yes
Cinnamic acid	148.05 g/mol	2	1	1.90	0	Yes
Caffeic acid	180.16 g/mol	4	3	0.70	0	Yes
Vanillic acid	168.15 g/mo	4	2	0.73	0	Yes
Quinic acid	192.17 g/mol	4	2	−2.14	0	Yes
Ferulic acid	194.18 g/mol	4	2	1.00	0	Yes
Di‐hydroferulic acid	196.20 g/mol	5	3	0.16	0	Yes
Caffeic acid 3‐glucoside	342.30 g/mol	9	6	−1.63	1 NHorOH > 5	Yes
Chlorogenic acid	354.31 g/mol	9	6	−1.05	1 NHorOH > 5	Yes
2‐(Glucosyloxy)‐4‐methoxy‐cinnamic acid	356.32 g/mol	9	5	−1.38	0	Yes
Flavonoids						
Apigenin	270.24 g/mol	5	3	0.52	0	Yes
Naringenin	272.25 g/mol	5	3	0.71	0	Yes
Kaempferol	286.24 g/mol	6	4	−0.03	0	Yes
Eriodictyol	288.25 g/mol	6	4	0.16	0	Yes
Catechin	290.27 g/mol	6	5	0.24	0	Yes
Epicatechin	290.27 g/mol	6	5	0.24	0	Yes
Quercetin	302.24 g/mol	7	5	−0.56	0	Yes
Isorhamnetin	316.26 g/mol	7	4	−0.31	0	Yes
Afzelin	432.38 g/mol	10	6	−1.34	1 NHorOH > 5	Yes
Quercitrin	448.38 g/mol	11	7	−2.10	2 NorO > 10, NHorOH > 5	No
Kaempferol 7‐O‐glucoside	448.38 g/mol	11	7	−1.84	2 NorO > 10, NHorOH > 5	No
Hyperoside	464.38 g/mol	12	8	−2.59	2 NorO > 10, NHorOH > 5	No
Rutin	610.52 g/mol	16	10	−3.89	3 MW > 500, NorO > 10, NHorOH > 5	No
Narcissin	624.54 g/mol	16	9	−3.69	3 MW > 500, NorO > 10, NHorOH > 5	No
Quercetin 3‐O‐(6″‐malonyl‐glucoside) 7‐O‐glucoside.	712.56 g/mol	20	11	−4.71	3 MW > 500, NorO > 10, NHorOH > 5	No
Quercetin 3‐O‐rhamnosyl‐(1‐>2)‐rhamnosyl‐(1‐>6)‐glucoside	756.66 g/mol	20	12	−5.22	3 MW > 500, NorO > 10, NHorOH > 5	No
Coumarins						
Coumarin	146.14 g/mol	2	0	1.65	0	Yes
Herniarin	176.17 g/mol	3	0	1.34	0	Yes
*Trans*‐o‐Coumaric acid 2‐glucoside	326.30 g/mol	8	5	−1.12	0	Yes
Taxifolin 7‐glucoside	466.39 g/mol	12	8	−2.67	2 violations: NorO > 10, NHorOH > 5	No
Anthocyanins						
Procyanidin B1	578.52 g/mol	12	10	−0.26	3 violations: MW > 500, NorO > 10, NHorOH > 5	No
Cyanidin 3,5‐diglucoside	611.53 g/mol	16	11	−3.82	3 violations: MW > 500, NorO > 10, NHorOH > 5	No
Cyanidin 3‐sambubioside	616.95 g/mol	15	10	−3.08	3 violations: MW > 500, NorO > 10, NHorOH > 5	No
Cyanidin 3,5‐diglucoside dimer	773.19 g/mol	16	11	−3.82	3 violations: MW > 500, NorO > 10, NHorOH > 5	No

**FIGURE 1:**
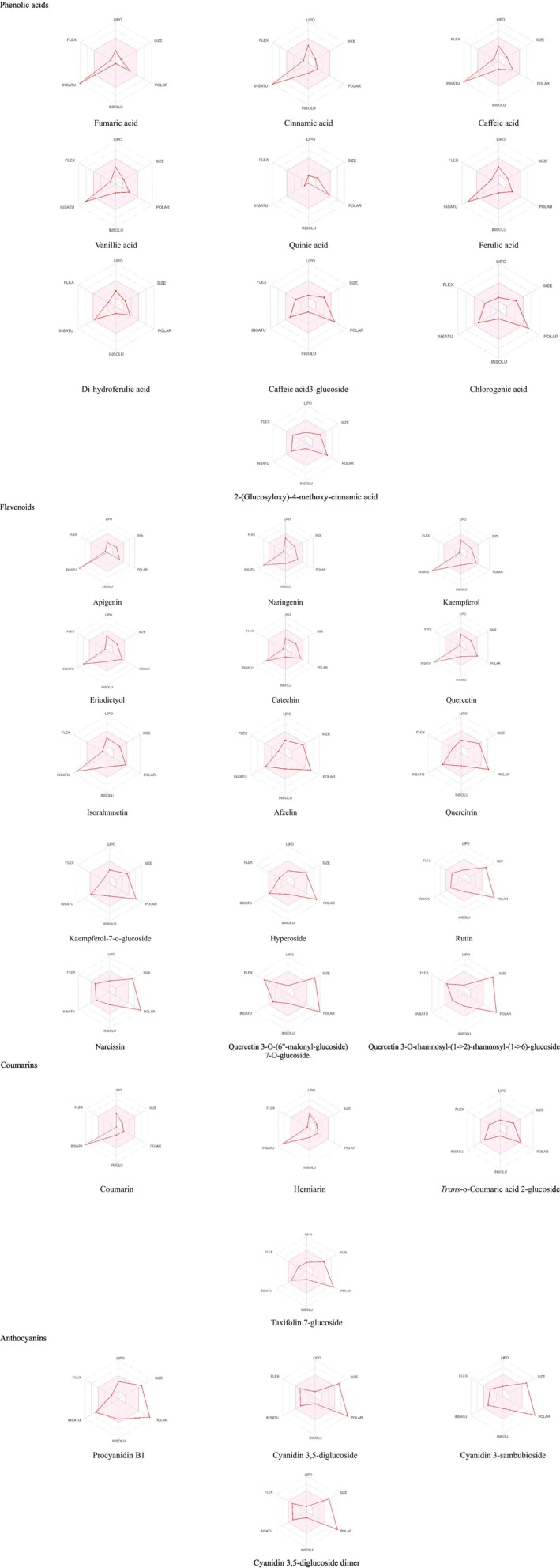
Radar plot technique for the major classes identified where: POLAR, LIPO, INSOLU, and IN‐SATU, stand for the compounds' polarity, lipophilicity, solubility, flexibility, and saturation on the radar map. The ideal range for every molecular attribute is shown by the pink region. Saturation: Carbon proportion in the sp3 hybridization > 0.25; polarity: TPSA between 20 and 130 Å; flexibility: < 9 rotatable bonds; solubility: Log S < 6; sizes: MW between 150 and 500 g/mol. XLOGP3 falls between −0.7 and + 5.0 for lipophilicity.

As observed in Figure 1, a metabolite is deemed drug‐like if its radar plot completely occupies the pink region as it represents the ideal range for each property: polarity: TPSA between 20 and 130 Å2, size: MW between 150 and 500 g/mol, solubility: log S not higher than 6, saturation: fraction of carbons in the sp^3^ hybridization not less than 0.25, and flexibility: no more than nine rotatable bonds. Quinic acid, ferulic acid, 2‐(Glucosyloxy)‐4‐methoxy‐cinnamic acid, afzelin, and trans‐o‐coumaric acid 2‐glucoside exhibited optimal profiles with all the six parameters located in the pink area; thus, they are considered good oral drug candidates and therefore could be considered for further studies. Notably, the rest of the identified compounds revealed deviation from the pink area in just one or two parameters. On the one hand, fumaric acid, cinnamic acid, caffeic acid, vanillic acid, apigenin, naringenin, kaempferol, eriodictyol, catechin, epicatechin, quercetin, isorhamnetin, coumarin, and herniarin displayed INSATU parameter deviancy. On the other hand, caffeic acid 3‐glucoside, chlorogenic acid, quercitrin, hyperoisde, kaempferol‐3‐glucoside, and taxifolin‐7‐glucoside were side‐shoots of the vertex in the polarity parameter. Regarding the size parameter, a clear deviation was observed in all the anthocyanin glycosides and 4 of the flavonoid glycosides, namely rutin, naricissin, Quercetin 3‐O‐(6″‐malonyl‐glucoside) 7‐O‐glucoside, and Quercetin 3‐O‐rhamnosyl‐(1‐>2)‐rhamnosyl‐(1‐>6)‐glucoside.

Poor pharmacokinetics and bioavailability are major contributing factors to many drug development failures, in addition to efficacy and toxicity. At different phases of the drug development process, it is essential to estimate two pharmacokinetic behaviors: brain permeability and gastrointestinal absorption. To achieve this, an accurate predictive model called the BOILED‐Egg is developed. It computes the lipophilicity and polarity of tiny compounds.

**FIGURE 2:**
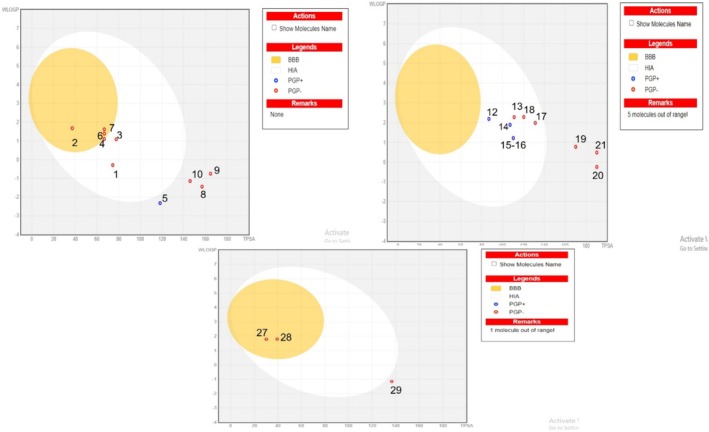
Boiled Egg method for the evaluation of the major classes of compounds. N.B. some compounds were located out of the range as they did not reach the threshold TPSA 201.28Å2.

The BOILED‐Egg model has proven to be a valuable tool for understanding and effectively translating molecular design in numerous drug discovery contexts (Figure 2). It makes it easier to intuitively evaluate brain penetration (BBB) (the yolk) and passive gastrointestinal absorption (HIA) (egg white). The term “gray region” refers to the physicochemical zone that contains metabolites anticipated to have substantial intestinal absorption. Furthermore, the software anticipated most compounds as non‐substrates (PGP−) of the permeability glycoprotein (PGP) being seen in red circles. Contrarily, only quinic acid, naringenin, eriodictyol, catechin, and epicatechin were shown as blue circles corresponding to a substrate (PGP +) of glycoprotein permeability. The threshold area (Daina and Zoete 2016) (TPSA 201.28 Å2) was not achieved by anthocyanins glycosides and 5 of the flavonoid glycosides, namely hyperoside, rutin, narcissin, Quercetin 3‐O‐(6″‐malonyl‐glucoside) and Quercetin 3‐O‐rhamnosyl‐(1‐>2)‐rhamnosyl‐(1‐>6)‐glucoside.

### 3.8 Molecular Docking Results

**FIGURE 3:**
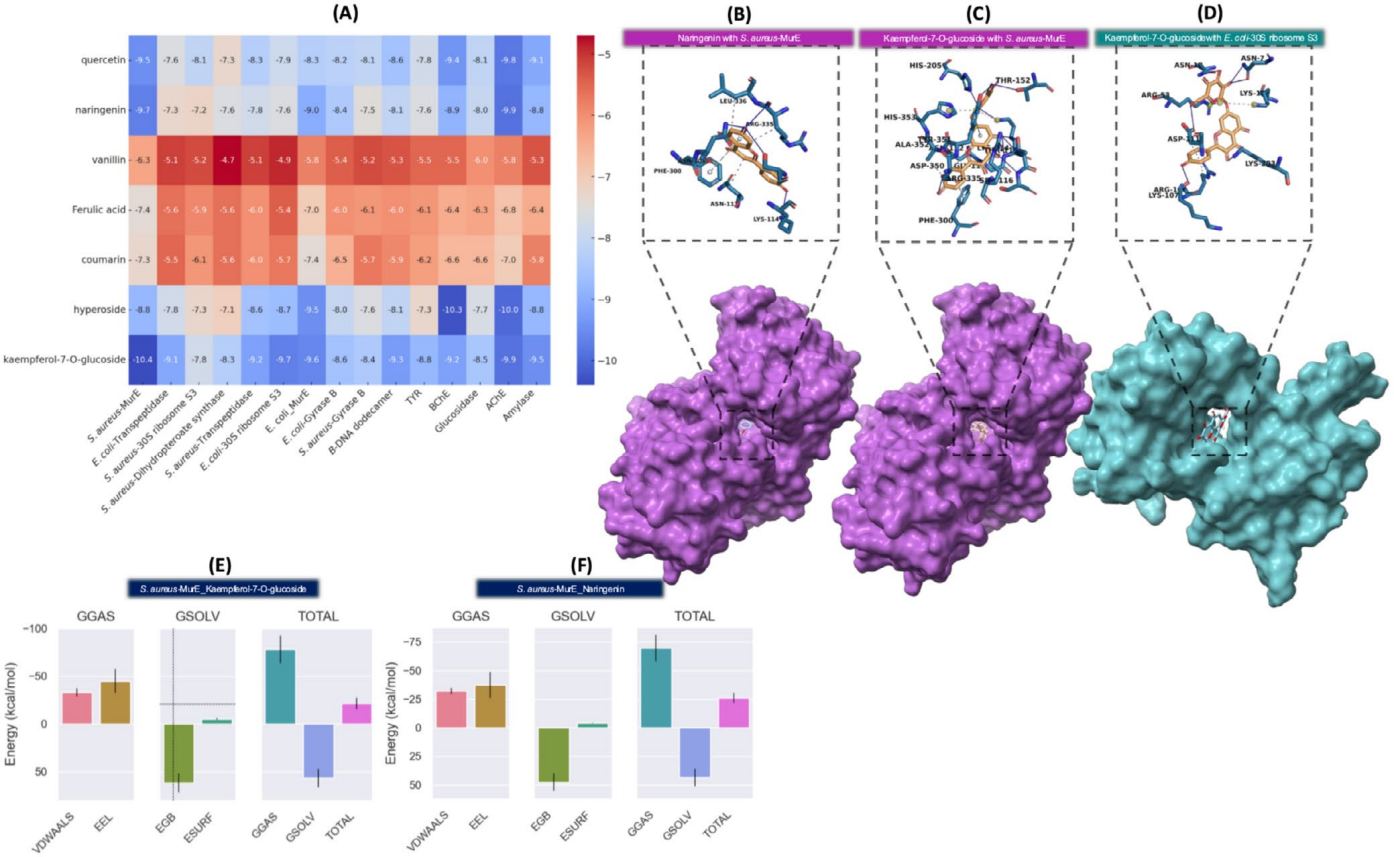
A comprehensive analysis of the binding interactions between enzymes/proteins and the selected compounds, along with MM/PBSA binding free energy calculations: (A) Graphical representation of docking scores for relevant proteins and enzymes. (B) Molecular interaction analysis of naringenin with *S. aureus* ‐MurE. (C) Molecular interaction analysis of kaempferol‐7‐O‐glucoside with *S. aureus* ‐MurE. (D) Molecular interaction analysis of kaempferol‐7‐O‐glucoside with *E. coli* ‐30S ribosome S3. (E) MM/PBSA binding free energy calculations of the S. aureus‐MurE_Kaempferol‐7‐O‐glucoside. (F)MM/PBSA binding free energy calculations of the S. aureus‐MurE_Naringenin.

This study employed a comprehensive evaluation approach to investigate the anti‐microbial and enzyme‐targeting properties of compounds identified in *P. mahaleb* against target‐related proteins and enzymes. The chemical profiling revealed a significant number of bioactive compounds, including quercetin, naringenin, vanillin, ferulic acid, coumarin, hyperoside, and kaempferol‐7‐O‐glucoside, against standard enzyme targets, including AChE, amylase, BChE, glucosidase, and TYR, *S. aureus* and *E. coli* related target 30S ribosome, dihydropteroate synthase, gyrase B, muramyl ligase E, transpeptidase, and B‐DNA dodecamer. Chemical profiling of these ligands revealed their potential bioactive nature, prompting an exploration of their binding modes, free energies of association, RMSD values, and hydrogen bond interactions. Figure 3 provides a comprehensive overview of all compounds with binding energies, while Table 9 focuses specifically on those compounds with binding energies equal to or less than −9 kcal/mol. The computational results revealed a wide range of binding energies from −10.4 to −3.9 kcal/mol and RMSD values from 0.2 to 8.3 Å. These results underscore the distinct affinity and conformational stability of each compound for different protein targets (Figure 3A, Table 9).

**TABLE 9:** The docking score (kcal/mol) and interacting residues of the enzyme and protein.

Compound	Target	PDB ID	Binding energy	RMSD	Interaction		Binding site
					Type	Number	
Quercetin	BChE	3DJY	−9.4	0.66	Hbond	6	ASN A:68;ASP A:70;ASP A:70;TRP A:82;TYR A:332;HIS A:438
Hyperoside	BChE	3DJY	−10.3	0.78	Hbond	7	ASP A:70;GLY A:115;GLY A:115;TYR A:128;ALA A:199;TYR A:332;HIS A:438
Kaempferol‐7‐O‐glucoside	BChE	3DJY	−9.2	0.55	Hbond	9	ASN A:68;GLY A:116;GLU A:197;ALA A:199;ALA A:199;GLU A:276;GLU A:276;PRO A:285;HIS A:438
Quercetin	AChE	2Y2V	−9.8	0.49	Hbond	6	ASP A:74;ASN A:87;ASN A:87;SER A:125;TYR A:133;GLU A:202
Naringenin	AChE	2Y2V	−9.9	0.96	Hbond	3	GLY A:121;GLY A:122;TYR A:337
Hyperoside	AChE	2Y2V	−10.0	1.08	Hbond	15	ASP A:74;TRP A:86;GLY A:121;GLY A:122;TYR A:124;TYR A:124;SER A:125;TYR A:133;TYR A:133;GLU A:202;PHE A:295;ARG A:296;ARG A:296;TYR A:341;HIS A:447
Kaempferol‐7‐O‐glucoside	AChE	2Y2V	−9.9	1.07	Hbond	5	TYR A:72;ASP A:74;TYR A:124;SER A:125;PHE A:295
Quercetin	Amylase	2QV4	−9.1	1.04	Hbond	6	GLN A:63;ARG A:195;ARG A:195;ASP A:197;HIS A:299;ASP A:300
Kaempferol‐7‐O‐glucoside	Amylase	2QV4	−9.5	0.04	Hbond	6	ASN A:53;GLN A:63;THR A:163;ARG A:195;GLU A:233;ASP A:300
Quercetin	*S. aureus‐*MurE	4C13	−9.5	0.56	Hbond	12	THR A:111;GLY A:113;LYS A:114;LYS A:114;THR A:115;THR A:115;THR A:115;SER A:116;HIS A:205;ARG A:335;HIS A:353;GLY A:357
Naringenin	*S. aureus‐*MurE	4C13	−9.7	1.03	Hbond	4	LYS A:114;SER A:116;ASN A:301;ARG A:335
Kaempferol‐7‐O‐glucoside	*S. aureus‐*MurE	4C13	−10.4	0.51	Hbond	11	GLY A:113;LYS A:114;LYS A:114;THR A:115;THR A:115;SER A:116;THR A:152;HIS A:205;ARG A:335;ASP A:350;TYR A:351
Kaempferol‐7‐O‐glucoside	*E. coli‐*Transpeptidase	6NTW	−9.1	1.07	Hbond	7	GLN A:216;ALA A:330;SER A:385;ASP A:520;ASP A:520;ARG A:522;TYR A:575
Kaempferol‐7‐O‐glucoside	*S. aureus* ‐Transpeptidase	5TW8	−9.2	0.39	Hbond	11	ASN A:72;GLU A:114;GLU A:114;GLU A:114;GLY A:181;GLY A:181;SER A:263;SER A:263;GLU A:297;ARG A:300;ARG A:300
Kaempferol‐7‐O‐glucoside	*E. coli* ‐30S ribosome S3	4 V53	−9.7	0.2	Hbond	6	ASN B:7;ASN B:7;ASN B:18;LYS B:107;ASP B:111;LYS B:203
Naringenin	*E. coli* ‐MurE	1E8C	−9.0	8.19	Hbond	5	SER B:283;GLY B:304;ASN B:307;ALA B:363;ALA B:370
Hyperoside	*E. coli* ‐MurE	1E8C	−9.5	8.39	Hbond	13	THR B:116;LYS B:119;SER B:184;SER B:184;ARG B:192;ARG B:192;ASP B:209;ASP B:209;HIS B:210;HIS B:210;TYR B:357;HIS B:359;LYS B:393
Kaempferol‐7‐O‐glucoside	*E. coli* ‐MurE	1E8C	−9.6	0.28	Hbond	14	ASN B:117;LYS B:119;THR B:120;THR B:120;THR B:142;THR B:142;THR B:157;HIS B:210;ARG B:341;ARG B:341;GLY B:386;ARG B:389;ARG B:389;ARG B:416
Kaempferol‐7‐O‐glucoside	B‐DNA dodecamer	1bna	−9.3	0.06	Hbond	0	

The identification of AChE, amylase, BChE, *E. coli* 30S ribosome S3, *E. coli* ‐MurE, *S. aureus‐MurE*, and *S. aureus* ‐Transpeptidase as key targets was based on their favorable docking parameters. For AChE, the compounds quercetin (RMSD = 0.49 Å, 6 H‐bonds, −9.8 kcal/mol), hyperoside (RMSD = 1.08 Å, 15 H‐bonds, −10.0 kcal/mol), and kaempferol‐7‐O‐glucoside (RMSD = 1.07 Å, 5 H‐bonds, −9.9 kcal/mol) interacted with critical residues, with ASP A:74 and SER A:125 being consistently involved in their binding. This finding underscores the importance of these residues in the catalytic pocket. A similar observation was made in the amylase assay, where quercetin (RMSD = 1.04 Å, 6 H‐bonds, −9.1 kcal/mol) and kaempferol‐7‐O‐glucoside (RMSD = 0. 04 Å, 6 H‐bonds, −9.5 kcal/mol) predominantly interacted around GLN A:63 and ASP A:300, with additional contacts at ARG A:195 and other nearby residues, suggesting a common interaction region. In the case of BChE, quercetin (RMSD = 0.66 Å, 6 H‐bonds, −9.4 kcal/mol), hyperoside (RMSD = 0.78 Å, 7 H‐bonds, −10.3 kcal/mol), and kaempferol‐7‐O‐glucoside (RMSD = 0.55 Å, 9 H‐bonds, −9.2 kcal/mol) all demonstrated interactions that centrally involved HIS A:438 along with additional residues such as ASP A:70 and GLU A:197, thereby highlighting common binding features despite similar energy profiles. For the *E. coli* 30S ribosome S3, kaempferol‐7‐O‐glucoside (RMSD = 0.20 Å, 6 H‐bonds, −9.7 kcal/mol) bound to a set (Figure 3D). This interaction was found to be specific, as it did not align with energy and hydrogen bond metrics alone. In the *E. coli* ‐MurE system, kaempferol‐7‐O‐glucoside demonstrated two distinct binding profiles. The first profile exhibited an RMSD of 0.28 Å, 14 H‐bonds, and − 9.6 kcal/mol, involving residues such as ASN B:117, LYS B:119, THR B:120/142, THR B:157, HIS B:210, and ARG B:341. The second profile exhibited an RMSD of 1. The interaction of quercetin (RMSD = 0.56 Å, 12 H‐bonds, −9.5 kcal/mol) with GLN A:216, ALA A:330, SER A:385, ASP A:520, ARG A:522, and TYR A:575 (RMSD = 0.7 Å, 7 H‐bonds, −9.1 kcal/mol) supports the hypothesis that common residue interactions are pivotal for effective binding. For *S. aureus* ‐MurE, quercetin (RMSD = 0.56 Å, 12 H‐bonds, −9.5 kcal/mol), naringenin (RMSD = 1.03 Å, 4 H‐bonds, −9.7 kcal/mol) (Figure 3B), and kaempferol‐7‐O‐glucoside (RMSD = 0. 51 Å, 11 H‐bonds, −10.4 kcal/mol) (Figure 3C) predominantly targeted LYS A:114, SER A:116, and ARG A:335, emphasizing these residues as key determinants of ligand binding in this enzyme. Finally, for *S. aureus* ‐Transpeptidase, kaempferol‐7‐O‐glucoside (RMSD = 0.39 Å, 11 H‐bonds, −9.2 kcal/mol) interacted with residues such as ASN A:72, GLU A:114, GLY A:181, SER A:263, GLU A:297, and ARG A:300, confirming a consistent binding pattern.

These findings strongly indicate that quercetin, naringenin, and kaempferol‐7‐O‐glucoside are potent inhibitors of *S. aureus* ‐MurE, with kaempferol‐7‐O‐glucoside also displaying high inhibition potential against *E. coli‐*30S ribosome S3 and *S. aureus* ‐Transpeptidase. Furthermore, quercetin and hyperoside exhibit strong interactions with AChE and BChE, reinforcing their enzymatic inhibition potential.

### 3.9 Binding Free Energy Analysis: MM/PBSA Results and Implications for Ligand Efficacy

In this study, molecular dynamics (MD) simulations were combined with molecular MM/PBSA calculations to assess the binding stability of several protein‐ligand complexes targeting *E. coli* and *S. aureus* proteins. The analysis incorporated key energy components, including van der Waals interactions (VDWAALS), electrostatic energy (EEL), polar solvation energy (EGB), surface tension (ESURF), gas‐phase energy (GGAS), solvation energy (GSOLV), and total binding free energy (TOTAL).

As illustrated in Table 10, the *E. coli* systems exhibited relatively moderate binding affinities. The *E. coli* ‐30S ribosome S3_Kaempferol‐7‐O‐glucoside and *E. coli* ‐MurE_Kaempferol‐7‐O‐glucoside complexes demonstrated total binding free energies of −10.21 and −12.89 kcal/mol, respectively. In contrast, the *S. aureus* ‐targeted complexes exhibited more favorable binding profiles, with the *S. aureus* ‐MurE_Quercetin complex displaying a total energy of −13.3 kcal/mol and the *S. aureus* ‐Transpeptidase_kaempferol‐7‐O‐glucoside complex exhibiting a total energy of −14.06 kcal/mol. It is noteworthy that the *S. aureus* ‐MurE_Kaempferol‐7‐O‐glucoside complex and the *S. aureus* ‐MurE_Naringenin complex were identified as the most promising candidates, with total binding free energies of −21.47 kcal/mol and −26.09 kcal/mol, respectively (Figure 3E,F). For instance, in the *S. aureus_MurE_Naringenin* complex, favorable van der Waals (−32.1 kcal/mol) and gas‐phase (−69.61 kcal/mol) energy contributions substantially outweighed the unfavorable polar solvation energy (47.69 kcal/mol), resulting in the most robust binding profile among the evaluated systems. A comparable outcome was observed in the *S. aureus* ‐MurE_kaempferol‐7‐O‐glucoside complex, which exhibited a robust van der Waals contribution of −33.09 kcal/mol and a gas‐phase contribution of −77.98 kcal/mol, thereby ensuring a stable binding interaction. The present findings underscore the pivotal role of non‐polar interactions and gas‐phase stabilization in determining ligand binding stability. Overall, while the *E. coli* ‐targeted complexes displayed relatively lower binding stability, the *S. aureus* ‐*MurE*_naringenin and *S. aureus* ‐MurE_kaempferol‐7‐O‐glucoside complexes emerged as the most promising inhibitor candidates based on their favorable binding free energy profiles and robust interaction stability, warranting further experimental validation.

**TABLE 10:** Binding free energy change and its components in different complexes.

Complex	Frames	VDWAALS	EEL	EGB	ESURF	GGAS	GSOLV	Total
*E. coli* ‐30S ribosome S3_Kaempferol‐7‐O‐glucoside	Average	−18.63	−19.56	30.78	−2.8	−38.2	27.98	−10.21
*E. coli* ‐MurE_Kaempferol‐7‐O‐glucoside	Average	−21.87	−46.88	59.85	−3.99	−68.76	55.86	−12.89
*S. aureus* ‐MurE_Quercetin	Average	−20.05	−32.4	42.24	−3.1	−52.44	39.14	−13.3
*S. aureus* ‐MurE_Kaempferol‐7‐O‐glucoside	Average	−33.09	−44.89	61.7	−5.18	−77.98	56.51	−21.47
*S. aureus* ‐Transpeptidase_Kaempferol‐7‐O‐glucoside	Average	−25.75	−31.93	47.84	−4.23	−57.67	43.62	−14.06
*S. aureus* ‐MurE_Naringenin	Average	−32.1	−37.52	47.69	−4.17	−69.61	43.52	−26.09

### 3.10 Molecular Dynamics Simulation

In the context of molecular dynamics simulation experiments, the focus has been on the *S. aureus* ‐MurE_Kaempferol‐7‐O‐glucoside and *S. aureus* ‐MurE_Naringenin complexes. The simulation time range extends from 0 to 100 ns.

**FIGURE 4:**
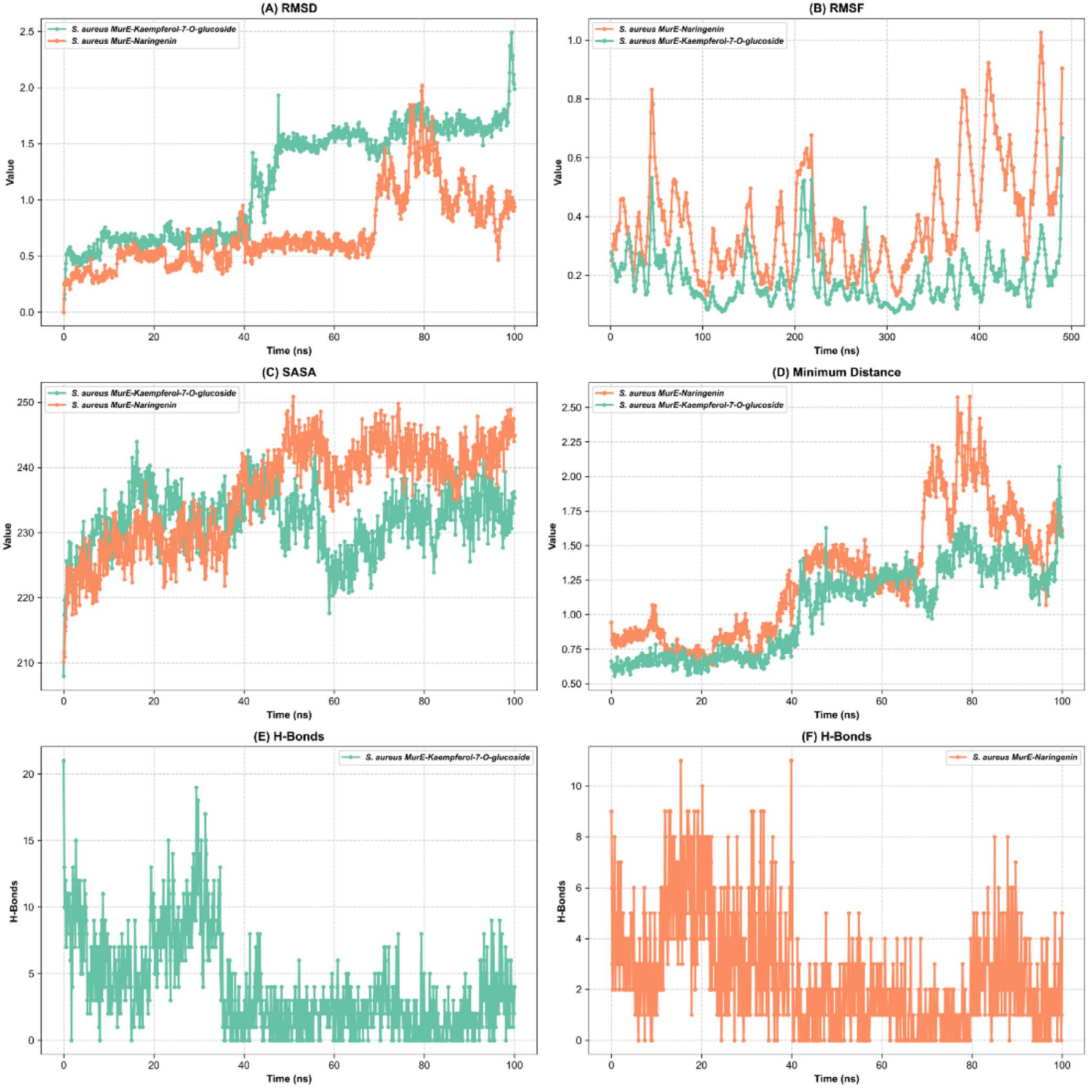
Molecular dynamics simulation results: (A) RMSD of *S. aureus* ‐MurE_Kaempferol‐7‐O‐glucoside and *S. aureus* ‐MurE_Naringenin. (B) RMSF of *S. aureus* ‐MurE_Kaempferol‐7‐O‐glucoside and *S. aureus* ‐MurE_Naringenin. (C) Solvent accessibility of *S. aureus* ‐MurE_Kaempferol‐7‐O‐glucoside and *S. aureus* ‐MurE_Naringenin. (D) Minimum distance of *S. aureus* ‐MurE_Kaempferol‐7‐O‐glucoside and *S. aureus* ‐MurE_Naringenin. (E) Hydrogen bonds of the *S. aureus* ‐MurE_Kaempferol‐7‐O‐glucoside complex. (F) Hydrogen bonds of the *S. aureus* ‐MurE_Naringenin complex.

The RMSD analysis revealed that the average RMSD value of 1.18 Å for *S. aureus* ‐MurE_Kaempferol‐7‐O‐glucoside was 0.44 Å for the 0–20 ns interval, 0.46 Å for the 20–40 ns interval, 0.50 Å for the 40–60 ns interval, and The *S. aureus* ‐MurE_Naringenin complex exhibited an average RMSD of 0.70 Å (0–20 ns: 0.26 Å, 20–40 ns: 0.33 Å, 40–60 ns: 0.37 Å, 60–80 ns: 0.31 Å, 80–100 ns: 0.73 Å) (Figure 4A). In the regional flexibility study, the Root Mean Square Fluctuation (RMSF) of the *S. aureus* ‐MurE_Kaempferol‐7‐O‐glucoside complex averaged 0.18 Å (min: 0.07 Å, max: 0.67 Å). The most fluctuating residues were observed at positions 490 (0.67 Å), 45 (0.53 Å), 218 (0.52 Å), 210 (0.52 Å), and 209 (0.52 Å). In contrast, the *S. aureus* ‐MurE_Naringenin complex exhibited an average RMSF of 0.38 Å (min: 0.13 Å, max: 1.03 Å), with the maximum fluctuations occurring at residues 467 (1.03 Å), 468 (0.98 Å), 466 (0.94 Å), 410 (0.92 Å), and 469 (0.92 Å) (Figure 4B). The Solvent‐Accessible Surface Area (SASA) analysis indicated that the *S. aureus* ‐MurE_Kaempferol‐7‐O‐glucoside complex had an average SASA of 231.7 nm^2^ (0–20 nm: 222.10 nm^2^, 20–40 nm: 223.37 nm^2^, 4 0–60 ns: 227.37 nm^2^, 60–80 ns: 230.20 nm^2^, 80–100 ns: 232.25 nm^2^). In contrast, the *S. aureus* ‐MurE_Naringenin complex exhibited an average SASA of 236.1 nm^2^ (0–2 0–20 ns: 219.39 nm^2^, 20–40 ns: 222.46 nm^2^, 40–60 ns: 224.27 nm^2^, 60–80 ns: 222.66 nm^2^, 80–100 ns: 237.28 nm^2^) (Figure 4C). In the minimum distance analysis, the *S. aureus* ‐MurE_Naringenin complex exhibited an average binding distance of 1.27 Å (0–20 ns: 0.81 Å, 20–40 ns: 0.82 Å, 40–60 ns: 0.85 Å, 60–80 ns: 0.85 Å, 80–100 ns: 1.31 Å). A separate distance analysis for the *S. aureus* ‐MurE_Kaempferol‐7‐O‐glucoside complex yielded an average distance of 1.04 Å (0–20 ns: 0.62 Å, 20–40 ns: 0.64 Å, 40–60 ns: 0.64 Å, 60–80 ns: 0.67 Å, 80–100 ns: 1.07 Å) (Figure 4D). Hydrogen bond (Hbond) analysis indicated that the *S. aureus* ‐MurE_Kaempferol‐7‐O‐glucoside complex formed an average of 4 Hbond (0–20 ns: 9, 20–40 ns: 10, 40–60 ns: 7, 60–80 ns: 5, 8 0–100 ns: 4), whereas the *S. aureus* ‐MurE_Naringenin complex averaged 3 Hbond (0–20 ns: 5, 20–40 ns: 4, 40–60 ns: 3, 60–80 ns: 3, 80–100 ns: 3) (Figure 4E,F).

Overall, these analyses demonstrate that both complexes exhibit time‐dependent structural adaptations in RMSD, RMSF, SASA, minimum distance, and hydrogen bond interactions over the 0–100 ns simulation period. The *S. aureus* _MurE_Kaempferol‐7‐O‐glucoside complex showed increasing RMSD values, indicating higher structural flexibility and residue fluctuations over time, which may affect its binding stability. In contrast, the *S. aureus* _MurE_Naringenin complex exhibited lower RMSD values and relatively stable hydrogen bonding, suggesting a more rigid and stable binding conformation. These findings highlight the dynamic differences between the two complexes, with Naringenin showing greater stability, but further validation is required to confirm its inhibitory potential.

## 4 Conclusion

In conclusion, the paper reported on the biological properties and chemical composition of the various parts of *P. mahaleb* . It was clear that the parts of the plant and the extraction solvents used affected the chemical profiles and biological characteristics. In general, the antioxidant activity of twig extracts was more potent than that of leaves and fruits. Methanol was more effective than water and ethyl acetate in terms of solvents. Phenolic acid, flavonoids, and coumarins were the most prevalent components of the chemical composition. In addition, the methanol extract of twigs exhibited significant antimutagenic potential, and none of the extracts that were tested were mutagenic. Consequently, *P. mahaleb* can serve as a valuable raw material for the development of effective agents that combat a variety of health conditions, such as Alzheimer's disease, cancer, diabetes, and microbial infections. However, it is strongly recommended that the individual components be isolated, their biological properties assessed, especially toxicological potential, and bioavailability/pharmacokinetic properties determined in future studies.

## Author Contributions

**Bayram Atasagun:** conceptualization (equal), investigation (equal), methodology (equal), writing – original draft (equal), writing – review and editing (equal). **Ahmet Uysal:** conceptualization (equal), data curation (equal), investigation (equal), methodology (equal), writing – original draft (equal), writing – review and editing (equal). **Noha Fathallah:** conceptualization (equal), investigation (equal), methodology (equal), writing – original draft (equal), writing – review and editing (equal). **Omayma Eldahshan:** conceptualization (equal), investigation (equal), methodology (equal), writing – original draft (equal), writing – review and editing (equal). **Abdel Nasser Singab:** data curation (equal), investigation (equal), methodology (equal), supervision (equal), writing – original draft (equal), writing – review and editing (equal). **Mehmet Veyis Cetiz:** data curation (equal), investigation (equal), methodology (equal), visualization (equal), writing – original draft (equal). **Gokhan Zengin:** conceptualization (equal), data curation (equal), investigation (equal), methodology (equal), writing – original draft (equal), writing – review and editing (equal).

## Conflicts of Interest

The authors declare no conflicts of interest.

## Data Availability

Data will be made available on request.
